# Estimating power in complex nonlinear structural equation modeling including moderation effects: The powerNLSEM R-package

**DOI:** 10.3758/s13428-024-02476-3

**Published:** 2024-09-20

**Authors:** Julien P. Irmer, Andreas G. Klein, Karin Schermelleh-Engel

**Affiliations:** Institute of Psychology, Department of Research Methods and Evaluation, Goethe University Frankfurt, Theodor-W.-Adorno-Platz 6, 60629 Frankfurt am Main, Germany

**Keywords:** powerNLSEM, moderation, mediation, power, LMS, product indicators, factor scores

## Abstract

The model-implied simulation-based power estimation (MSPE) approach is a new general method for power estimation (Irmer et al., 2024). MSPE was developed especially for power estimation of non-linear structural equation models (SEM), but it also can be applied to linear SEM and manifest models using the R package powerNLSEM. After first providing some information about MSPE and the new adaptive algorithm that automatically selects sample sizes for the best prediction of power using simulation, a tutorial on how to conduct the MSPE for quadratic and interaction SEM (QISEM) using the powerNLSEM package is provided. Power estimation is demonstrated for four methods, latent moderated structural equations (LMS), the unconstrained product indicator (UPI), a simple factor score regression (FSR), and a scale regression (SR) approach to QISEM. In two simulation studies, we highlight the performance of the MSPE for all four methods applied to two QISEM with varying complexity and reliability. Further, we justify the settings of the newly developed adaptive search algorithm via performance evaluations using simulation. Overall, the MSPE using the adaptive approach performs well in terms of bias and Type I error rates.

## Introduction

In recent years, complex structural equation models (SEM) have become prevalent, increasingly using models with nonlinear effects, indirect effects in mediator models, or moderated indirect effects in mediator models. While it is now routine for research proposals using classical statistical procedures (such as analysis of variance [ANOVA] or regression) to report power analyses to determine the required sample size, this has not been the case for linear and nonlinear SEM (here called NLSEM). While there are now both R packages and Shiny Apps available for analytical asymptotic power analysis for linear SEM (see Jak et al., 2021; Jobst et al., 2023; Moshagen & Erdfelder, 2016; Zhang & Yuan, 2018), this is not the case for nonlinear models, for which there only exist approaches for moderated regression models (see Baranger et al., 2022). Next to (asymptotic) analytical power estimation methods that enable the researcher to derive a required sample size before conducting a study, power estimation using a simulation for a predefined sample size is also possible (e.g., in M*plus*, Muthén & Muthén, 1998-2017).

Accurate estimation of statistical power is a crucial aspect of quantitative research. Before conducting a study, it is essential to determine whether the study has sufficient power to detect a certain effect within the statistical test (e.g., see Cohen, 1988, 1992). Statistical power refers to the probability of correctly rejecting the null hypothesis ($H_0$) in a sample when the alternative hypothesis ($H_1$) is true for the population given a certain significance level $\alpha$.

There are two main types of power analyses: global power analysis and local power analysis, depending on the specificity of the hypotheses being tested. Global power analysis deals with overall model characteristics, such as the overall model fit (Jobst et al., 2023). On the other hand, local power analysis does focus on the significance of individual parameters within a model. In this article, our emphasis lies on local power analysis for single parameters, whereas global power analysis relies on global measures of model fit. Unfortunately, global fit measures for NLSEM are only available for a limited class of SEM (see, e.g., Büchner & Klein, 2020). For local power analysis, it is crucial to ensure that the sample size (*n*) is sufficiently large to achieve a significant result with a reasonable probability, considering a specific expected effect size.

Expected effect size should ideally be derived from the existing literature (e.g., see Cohen, 1988, 1992). However, in statistical modeling scenarios with numerous parameters, deriving expected parameter values from the literature becomes challenging, particularly when the specific scenario of interest has not been previously investigated, making a-priori power analyses, power analyses conducted before a sample is collected, very difficult. In such cases, post-hoc or retrospective power analyses are often employed (Hoenig & Heisey, 2001; Jobst et al., 2023). Instead of using expected parameters prior to the collection of data in an a-priori power analysis, post-hoc power estimation methods rely on parameter estimates obtained from the collected sample. As a consequence of utilizing sample information, the resulting power values are a function of the *p*-value associated with a specific effect size being investigated (Hoenig & Heisey, 2001). In fact, the power of post-hoc power analyses is a monotonic function of the *p*-value. Consequently, retrospective power analyses are generally discouraged.

Instead of completely abandoning power analyses in such situations, researchers have proposed the use of Smallest Effect Size of Interest (SESOI, Anvari & Lakens, 2021). SESOI represents the minimal effect size that can still be deemed relevant within a specific research field. So instead of running a post-hoc power analysis, one can analyze the power for an SESOI even in scenarios in which the effect of interest was non-significant. Hence, for models with many parameters a SESOI for every parameter can be derived. This then enables the researcher to conduct a-priori power analyses or meaningful post-hoc power analyses after the sample has been collected (Anvari & Lakens, 2021).

Statistical power can be derived analytically for many test statistics by determining their (asymptotic) distribution under the alternative hypothesis. This can be done using software tools like G*Power (Faul et al., 2007, 2009), or packages such as pwr (Champely, 2020) and WebPower (Zhang & Yuan, 2018), which implement various basic statistical tests in R (R Core Team, 2023). However, in scenarios where there is no (asymptotic) analytical derivation of statistical power, such as models under distributional misspecification or limited information approaches, simulation-based procedures are utilized. Here, power is estimated by observing the relative frequency of a significant effect for a given sample size *n*.

In this article, we focus on a special class of NLSEM, namely models with quadratic and interaction effects (here called QISEM), which include models with single and multiple moderated mediation effects. For QISEM, power estimation is especially important as effect sizes for moderation (also called interaction) effects or quadratic effects are quite small in applied research (see, e.g., Chaplin, 1991, 2007). This is why smaller nonlinear effects have been analyzed in research concerned with QISEM (see, e.g., Brandt et al., 2014, 2020).

Although several R-packages and Shiny Apps have already been developed for linear SEM, there are only few developments for nonlinear models. These developments concentrate on moderated regression models (see Baranger et al., 2022; Shieh, 2009), hierarchical models with moderators (Dong et al., 2021) or latent models with categorical moderators (Donnelly et al., 2022). Measurement errors have not or only been indirectly considered (Baranger et al., 2022). For QISEM with continuous variables, no analytical approach has yet been proposed to estimate statistical power and, as a consequence, no implementation is available. This is due to the high complexity of the model and the higher order moments that are necessary to fit such types of NLSEM (e.g., Bollen, 1989). We note that the Monte Carlo procedure of M*plus* can be used to estimate power for single parameters in some NLSEM for a single sample size (Muthén & Muthén, 1998-2017, 2002), but an optimization routine to derive a required sample size for a given power rate is still missing.

Simulation-based power estimation methods, particularly for linear SEM, have become increasingly popular due to their ability to accommodate specific sample characteristics such as distributional misspecifications, especially in models lacking analytical (asymptotic) power solutions (e.g., Mulder, 2023; Wang & Rhemtulla, 2021). However, these methods typically rely on the selection of sample sizes, as the precision of power estimation is highest only for these specific sample sizes (see also Irmer et al., 2024). In contrast, the recently developed model-implied simulation-based power estimation (MSPE) approach by Irmer et al. (2024) offers a novel solution. The MSPE predicts statistical power by modeling the normality of parameter estimates by fitting a probit regression model to the significance decisions per simulated sample. The resulting power curve is then inverted to find the sample size necessary to yield a predefined power value by further taking the uncertainty of the fitting process directly into account. The use of a parametric model to predict power makes the MSPE more efficient compared to conventional power analysis that use point-wise estimates of power for selected sample sizes, as the MSPE economically uses the statistical information of all simulations describing the relation between power rate and sample size. Irmer et al. (2024) have shown that their approach is useful for a wide class of *M*-estimators (see, e.g., Wooldrige, 2010, Chapter12), which include (robust) maximum-likelihood (ML) linear SEM and QISEM using robust ML estimation based on the unconstrained product indicator approach (UPI; Kelava & Brandt, 2009; Marsh et al., 2004). In their illustration, Irmer et al. (2024) selected sample sizes that did not result in power rates larger than .99, as sample sizes drastically exceeding this threshold would require enormous replication counts to benefit from the fit of the probit regression model. Therefore, before the MSPE is generally applicable to several methods for estimating QISEM, the crucial choice of sample sizes needs to be resolved to ensure reliable estimates for required sample sizes.

Hence, the aims of this article are threefold. First, we propose an adaptive algorithm that automatically selects sample sizes to aid the prediction value of the MSPE, making the MSPE applicable without more technical requirements. The performance of the adaptive algorithm is compared to an a-priori selection in sample sizes, and optimal settings for the adaptive algorithm are derived. Second, we apply the MSPE to several QISEM and use the adaptive algorithm to select the necessary sample sizes. We discuss the influence of item level reliablity and model complexity for the latent moderated structural equations (LMS, Klein & Moosbrugger, 2000) approach, UPI, simple factor score regression, and scale regression, on statistical power. And, third, we provide a tutorial for using this new MSPE procedure including the adaptive algorithm in the selection of sample sizes in R (R Core Team, 2023) in the powerNLSEM package (Irmer, 2024). We end the article with a general discussion and practical recommendations when using the MSPE procedure with the powerNLSEM package.

## Model-implied simulation-based power estimation in QISEM

The model-implied simulation-based power estimation (MSPE) approach by Irmer et al. (2024) is a method that enables estimating the power of nonlinear effects in SEM using continuous latent variables. In contrast to the naïve approach in sample size selection (e.g., Mulder, 2023; Wang & Rhemtulla, 2021) that uses an ad-hoc selection of specific sample sizes with linear interpolation to show the relationship between the (estimated) statistical power and the sample size *n*, MSPE better approximates the continuous relationship between statistical power and sample size using a probit regression model. Before demonstrating MSPE for different QISEM, we provide a short explanation of the MSPE method.

### The MSPE method

**Fig. 1:**
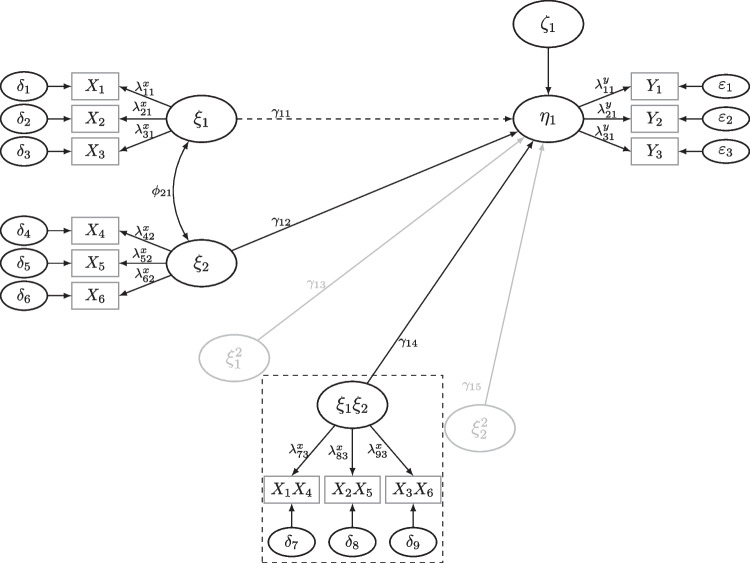
QISEM Including Moderation and Quadratic Effects.  *Note.* Grey effects represent effects not included in the simulation study. Mean structure and intercepts are not included in the figure. Dashed box shows product indicators formed within the unconstrained product indicator approach used to fit the QISEM

Let $\vartheta _0$ be the population parameter values set in a power analysis. For instance, in QISEM analysis $\vartheta _0$ contains factor loadings ($\lambda ^x,\lambda ^y$), measurement error variances ($\theta ^x,\theta ^y$), variance and covariance parameteres of the latent variables ($\phi ,\psi$), and regression parameters which consist of the linear, quadratic, and interaction effects ($\gamma$); see Fig. 1 or Appendix A. Then, for *R* samples of size $n_i$ for $i=1,\dots ,R$, we estimate $\vartheta _0$ by $\hat{\vartheta }_{n_i}$ and its corresponding standard errors. The power is estimated for a parameter of interest (POI) selected by the researcher.

Let the *j*-th parameter within $\vartheta _0$ be the POI $(\vartheta _{0,j})$ for the power analysis. $\vartheta _{0,j}$ is estimated by $\hat{\vartheta }_{n_i,j}$ with standard error $SE(\hat{\vartheta }_{n_i,j})$ within a sample of size $n_i$. Due to the asymptotic normality of the parameter estimates, the commonly used *z*-test can be applied to evaluate significance of single parameters:

$$ \mathcal {Z}_{n_i} = \frac{\hat{\vartheta }_{n_i,j}}{SE(\hat{\vartheta }_{n_i,j})}. $$

The *z*-value is compared to the $1-\alpha$ quantile $q_\text (1-\alpha )$ of the standard normal distribution as the significance test. Then for a one-sided *z*-test, let $\mathcal {S}_i$ be the significance decision of the one-sided test for $i=1,\dots ,R$. Here, $\mathcal {S}_i$ is 1 if the test examining the POI was significant and 0 else.

The MSPE utilizes the asymptotic normality property of the estimates $\hat{\vartheta }_{n_i}$ and the corresponding *z*-test by fitting a probit regression model with $\sqrt{n_i}$ as a predictor of the significance decisions $\mathcal {S}_i$ (Irmer et al., 2024). Hence, the power, defined as the probability of a significant effect, is modeled as a standard probit regression model with $\sqrt{n_i}$ as a predictor for $i=1,\dots ,R$

1 $$\begin{aligned} \mathbb {P}(\mathcal {S}_i = 1 | n_i, \beta _0, \beta _1) = \Phi (\beta _0 + \beta _1\sqrt{n_i}), \end{aligned}$$

where $\Phi$ is the cumulative density function of the standard normal distribution and $\beta _\text {probit}=(\beta _0,\beta _1)'$ are the parameters to be estimated by the probit regression fit to the significance decision. These coefficients have limits of

2 $$\begin{aligned} \beta _\text {probit}=\left( -q_\text (1-\alpha ),\frac{\left| \vartheta _{0,j}\right| }{\sqrt{\left( \mathcal {H}^ {-1} \mathcal {I}\mathcal {H}^ {-1} \right) _{j,j}}}\right) ', \end{aligned}$$

for large *n* and $R\rightarrow \infty$, where $\mathcal {H}$ is the expected Hessian of objective function, $\mathcal {I}$ is the variance of the score function of the objective function (i.e., the expected Fisher information in ML-estimation), and $\left( \mathcal {H}^ {-1} \mathcal {I}\mathcal {H}^ {-1} \right) _{j,j}$ is the *j*-th diagonal element of the asymptotic covariance matrix of the estimated parameter vector $\hat{\vartheta }_{n_i}$ (White, 1982; Huber, 1967), which has often been called the sandwhich estimator for standard errors (see, e.g., Klein & Muthén, 2007; Savalei, 2014). The square root of this element divided by $\sqrt{n_i}$ gives the standard error. These probit regression parameters are used to calculate the required sample size $N_\alpha$ for a predefined power rate $\rho$ (Irmer et al., 2024)

3 $$\begin{aligned} N_\alpha&\approx \left\lceil \left( \frac{\Phi ^ {-1} (\rho ) -\beta _0}{\beta _1}\right) ^2\right\rceil =&\nonumber \\&\left\lceil \Bigg (\Phi ^ {-1} (\rho ) + q_\text (1-\alpha ) \Bigg )^2 \cdot \left( \frac{|\vartheta _{0,j}|}{\sqrt{\left( \mathcal {H}^ {-1} \mathcal {I}\mathcal {H}^ {-1} \right) _{j,j}}}\right) ^{-2}\right\rceil , \end{aligned}$$

where $\lceil \cdot \rceil$ rounds up to the next integer.

When the probit regression coefficients are estimated, they underlie sample variability. This needs to be considered, as it has to be ensured that the computed sample size yields the required power rate $\rho$. This is done by the computation of the required sample size that corresponds to the lower bound of the $1-\alpha _\rho$ confidence interval around the estimated power rates $\hat{\rho }$ equaling the desired power rate. $\hat{\rho }_{\rho _\text {lb} = \rho }$ is the smallest estimated power rate that has a Type I error of $\alpha _\rho /2$ and can be used to calculate

4 $$\begin{aligned} N_{\alpha ,\text {lb}}&\approx \left\lceil \left( \frac{\Phi ^ {-1} \left( \hat{\rho }_{\hat{\rho }_\text {lb} = \rho }\right) -\beta _0}{\beta _1}\right) ^2\right\rceil , \end{aligned}$$

which is larger or equal to $N_\alpha$ with probability $\approx 1-\alpha _\rho /2$ (Irmer et al., 2024). The sample sizes then are estimated by replacing the coefficients $\beta _0,\beta _1$ by their estimates $\hat{\beta }_0,\hat{\beta }_1$. Pseudo code for the MSPE is given in Algorithm 1.

**Algorithm 1:**
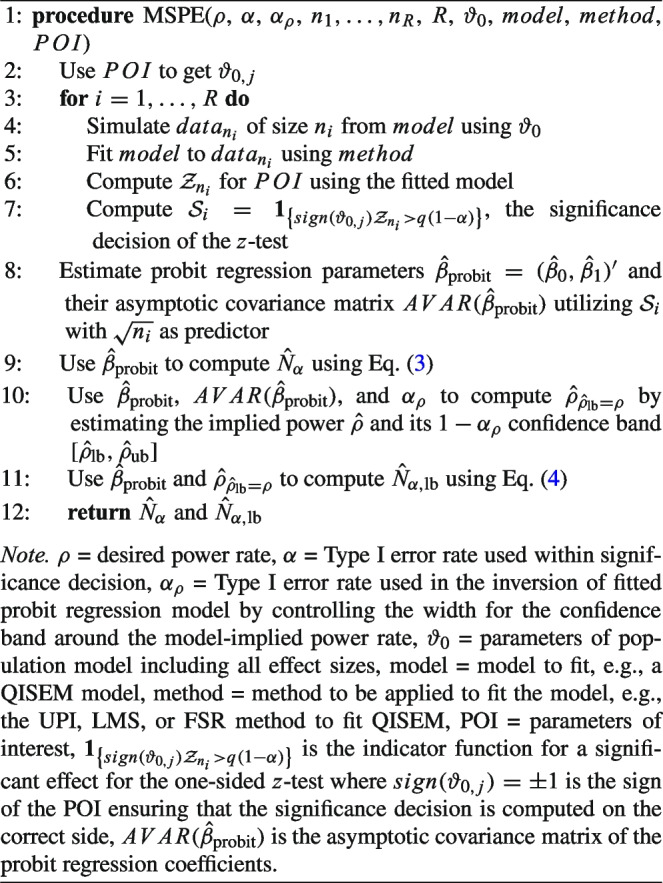
Pseudo code for the MSPE.

For the MSPE to be applicable, a selection of sample sizes in necessary. Irmer et al. (2024) used an a-priori selection of samples sizes that correspond to power rates $\rho \le .99$. However, when applying the MSPE to new power scenarios these sample sizes are not know. Hence, a selection has to be made. In this paper we propose an *adaptive* algorithm, for the selection of sample sizes that evaluates the MSPE several times and then selects new sample sizes to ensure that the required sample size can be estimated with high precision. This adaptive algorithm is implemented in the powerNLSEM package (Irmer, 2024). Simulation studies examining the performance of the adaptive algorithm in comparison to an a-priori choice of sample sizes are presented after the tutorial. Detailed description and pseudo code are also given after the tutorial.

## The powerNLSEM package: A description and tutorial

In this section, we present the powerNLSEM R-package (Irmer, 2024) for determining the required sample size given a specific power in QISEM and to estimate power using the adaptive approach to MSPE. We begin with briefly outlining the methods used in the package to fit QISEM: latent moderated structural equations (LMS, Klein & Moosbrugger, 2000), a simple factor score regression (FSR) approach as proposed by Ng & Chan (2020), the unconstrained product indicator approach (UPI, Marsh et al., 2004; Kelava & Brandt, 2009), and a scale regression approach using single indicator scale means per latent variables as proxies for latent variables in a path model. We apply these methods to a simple QISEM model first (see Fig. 1). Later we apply these methods to a more complex SEM including moderated mediation effects, as illustrated in Fig. 6.

The structural model of the simple QISEM is given by

5 $$\begin{aligned} \eta _1&= \alpha _1 + \gamma _{11}\xi _1 + \gamma _{12}\xi _2 + \gamma _{13}\xi _1^2 \nonumber \\&\quad + \gamma _{14}\xi _1\xi _2 + \gamma _{15}\xi _2^2 + \zeta _1. \end{aligned}$$

In this model, the intercept of $\eta _1$ is denoted by $\alpha _1$, the linear effects of $\xi _1$ and $\xi _2$ on $\eta _1$ are $\gamma _{11}$ and $\gamma _{12}$, the quadratic effects of $\xi _1^2$ and $\xi _2^2$ on $\eta _1$ are denoted by $\gamma _{13}$ and $\gamma _{15}$, respectively, and the moderation (interaction) effect between $\xi _1$ and $\xi _2$ on $\eta _1$ is denoted by $\gamma _{14}$. If we fix the quadratic effects to zero and select $\xi _2$ as the moderator (this choice is arbitrary and cannot be made statistically, as the model is symmetric), then $\gamma _{14}$ quantifies the extend to which the slope of $\xi _1$ on $\eta _1$ depends on the values of the moderator $\xi _2$.

### Methods for the estimation of quadratic and interaction SEM within the powerNLSEM package

Next to single indicator moderated regression approaches (Cheung & Lau, 2017), i.e., SR, the latent moderated structural equations (LMS, Klein & Moosbrugger, 2000) approach and the (unconstrained) product indicator approach (UPI, Kelava & Brandt, 2009; Marsh et al., 2004) are the most widely used approaches to fitting QISEM in applied research (Aytürk et al., 2020). Further, simple factor score approaches (as studied by Ng & Chan, 2020) are easy to use for applied researchers.

LMS is a distribution analytical approach, which can be viewed as the ML approach to QISEM (Schermelleh-Engel et al., 1998; Klein & Moosbrugger, 2000). It relies on the multivariate normality of the latent predictor variables, the residuals and measurement errors, while the model implied non-normality of the latent and manifest dependent variables is included in the model by a mixture of distributions. All coefficients are estimated simultaneously by maximizing the log-likelihood of the model using the expectation maximization (EM, Dempster et al., 1977) algorithm. The Fisher information is used to derived ML standard errors (Schermelleh-Engel et al., 1998; Klein & Moosbrugger, 2000). Most research using LMS utilized M*plus* (Muthén & Muthén, 1998-2017), however, an R implementation can be found in the nlsem package (Umbach et al., 2017). Robust standard errors can be used in M*plus* via the use of the sandwich estimator (see, e.g., Huber, 1967; White, 1982; Wooldrige, 2010). We fit QISEM using LMS with M*plus* and make use of the MplusAutomation (Hallquist & Wiley, 2018) R package to call the method. Hence, M*plus* is only called implicitly, but an installation is required.

The UPI approach employs products of indicators as measurements for the nonlinear terms in Quadratic and Interaction Structural Equation Models (QISEM). In this approach, the factor loadings and latent variances of the nonlinear terms are expressed as functions of the linear model parameters. While earlier product indicator approaches included nonlinear constraints in the model (see Jöreskog & Yang, 1996; Kenny & Judd, 1984), the unconstrained approach fits all parameters freely, thereby enhancing robustness to non-normality and simplifying model formulation by eliminating the need for nonlinear constraints (Marsh et al., 2004; Kelava & Brandt, 2009). The selection of product indicators is not free of ambiguity (Foldnes & Hagtvet, 2014) as pairs can be matched or all combination can be used. In matching generally higher reliability pairs are preferred (Wu et al., 2013), although matching may influence parameter estimation in an arbitrary way (Foldnes & Hagtvet, 2014). Various centering approaches can be applied during the formation of product indicators to stabilize parameter estimation. These include the centered residual approach (Little et al., 2006), the mean-centered approach (Marsh et al., 2007), and the double mean-centered approach (Lin et al., 2010), which eliminates the mean structure while providing optimal estimation of structural parameters. In the powerNLSEM package, product indicators are computed using the semTools package (Jorgensen et al., 2020), which supports all centering strategies and a matching procedure, as well as the computation of all combinations of product indicators. Models are fitted using lavaan (Rosseel, 2012) with robust standard errors.

Simple factor score approaches estimate factor scores in a first step and then use the factor scores as well as functions of the factor scores in a path model. Devlieger and Rosseel (2017) showed via simulation that factor scores can be used to fit linear SEM and Ng and Chan (2020) proposed to use them to fit QISEM with best performance for the SL method (named after Skrondal & Laake, 2001). In the SL method the regression factor scores (Thomson, 1934; Thurstone, 1935) are used to estimate the independent variables and Bartlett (1937) factor scores are used to estimate the dependent variables of the model (Skrondal & Laake, 2001). Nonlinear terms are then constructed similarly to moderated or regression analysis (Ng & Chan, 2020). However, it is important to note that factor scores are merely estimates of the latent variables and, therefore, subject to distortion by measurement error, rendering them biased, and the standard errors do not directly account for the estimation process of the factor scores. This stands in contrast to the approach of Wall and Amemiya (2000, 2003), which is unbiased and efficient but challenging to implement. As the number of measurements increases, the estimated factor scores tend to approach the underlying latent variables in mean square (Krijnen, 2006b, a). Consequently, for larger numbers of measurements, the residual variance of the predicted factor scores diminishes, and parameters within the QISEM estimation are assumed to converge toward the population values (Skrondal & Laake, 2001), as the conditional expectation converges toward the underlying conditional expectation (Grønneberg & Irmer, 2024). Estimation of factor scores and corresponding path models is conducted using lavaan (Rosseel, 2012) with robust standard errors.

For SR, the scale means per latent variable are used as single indicators for each latent variable in fitting a path model, where nonlinear terms are formed using products of the centered scale means. These path models are fitted using lavaan (Rosseel, 2012) with robust standard errors. Cheung and Lau (2017) indicate that single indicator approaches are widely used due to their simplicity. However, we note that the SR approach does not adequately model measurement errors and is not recommended to be used.

LMS is a distribution-analytic approach and, hence, suffers from distributional misspecification. However, LMS has shown to be robust to moderate skewness of predictor variables in simulation studies (e.g., Brandt et al., 2014). The UPI and FSR approach using robust standard errors, do not have such strong distributional assumptions and are expected to have little or no bias under distributional misspecifications. Irmer et al. (2024) showed that MSPE for QISEM with non-normal predictors performed well when fitted with UPI, resulting in low bias, low RMSE, and low Type I error rates with respect to the required sample sizes. In these simulations, the UPI method showed only slightly inflated Type I error rates in the significance decision of about $6.5\%$. This is in line with previous simulation studies (e.g., Brandt et al., 2014).

### Steps of the powerNLSEM package

In order to estimate power of certain coefficients with the powerNLSEM package using MSPE, the user needs to set all required parameters within the model to specific values. This can be challenging and we suggest to use SESOIs when theoretical values cannot be derived. Further, we suggest to run several power analyses for different values that are not of main interest, such as factor loadings, which can have large influence on the required sample sizes. For instance, the factor loadings and residual variances determine the reliability of the items used per latent variables and inflict strong effects on the power of parameters estimates of the structural model (see upcoming simulation studies).

The model is formulated in lavaan-notation (see documentation of the package, Rosseel, 2012). The user then needs to specify for which estimation method for QISEM power should be estimated, how many repetitions *R* of the model should be fit and which search algorithm to use (adaptive or an ad-hoc selection of sample sizes). The adaptive algorithm automatically selects sample sizes for an optimized estimation of power (see Algorithm 3 and Fig. 3), while the ad-hoc algorithm (see Algorithm 2) requires a selection of sample sizes by the user using prior knowledge on power. The parameters of interest (POI) need to be selected. Further, the Type I error rate $\alpha$ for the significance decision, the desired power rate $\rho$ and the Type I error for the estimation of the power $\alpha _\rho$ can be specified. All (hyper-)parameters within the adaptive algorithm can be altered by the user. Further, we can specify which test to use (one-sided vs two-sided) and what power model to use (probit, Wald, or logit).

QISEM is simulated with the equation by equation approach. This means that each dependent variable is simulated constructively using the variables that it depends on. Predictors and measurement errors are multivariate normally distributed and the endogenous variables are model-implied non-normally distributed (Jöreskog & Yang, 1996). Non-normal predictors or measurement errors are not implemented yet.

#### Model formulation for simple latent moderation model

We translate the simple latent moderation model of Eq. (5), depicted in Fig. 1, into lavaan syntax and select the parameter values to yield high reliability (see Appendix A for more details). The moderation between $\xi _1$ and $\xi _2$ is set to $\gamma _{14}=.1$ and the residual variance of $\eta _1$ ($\psi _{11}=\mathbb {V}ar \, [\zeta _1]$) is set to 0.5975, which corresponds to a variance increment of the interaction effect of $1.25\%$, i.e., the moderation itself explains $1.25\%$ of the total variance of $\eta _1$, the dependent variable. This is a realistic effect size (e.g., Chaplin, 2007).

In this example, we are interested in the moderation effect in our model, hence, the parameter of interest (POI) is $\gamma _{14}$ of Eq. (5). For every POI we evaluate the significance of the parameter. However, the required sample size that is computed will yield a power rate of $\ge .8$ for all parameters. Hence, the parameter resulting in the lowest power per sample size will decide the final required sample size. The model needs to be written in lavaan (Rosseel, 2012) syntax given as a string (i.e., in quotation marks "..."). 
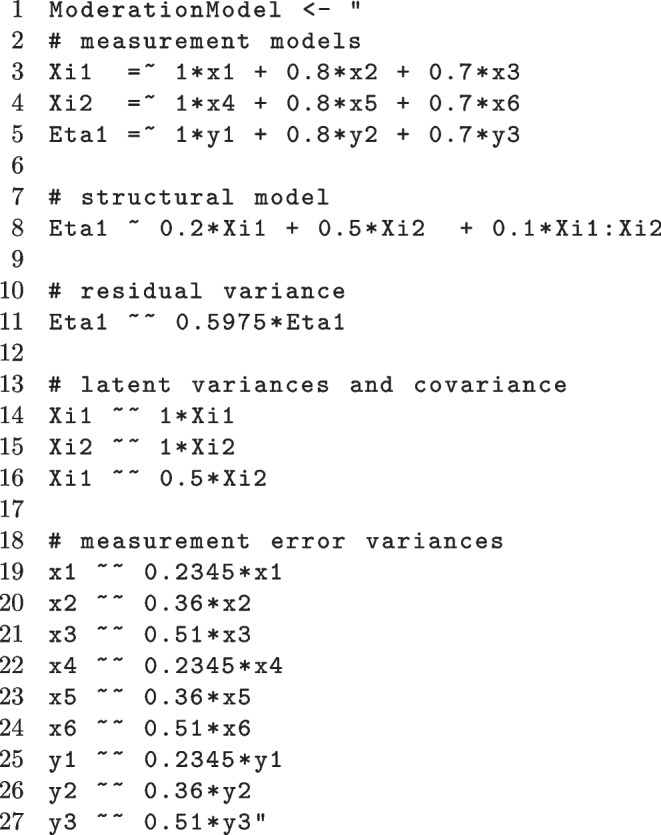


In this example, ModerationModel is the model name. Comments within the model formulation are initiated with a hash symbol (#). The latent variables Xi1, Xi2, and Eta1 are measured each by three indicators x1, x2, x3, x4, x5, x6, and y1, y2, y3, respectively (“measured by” is denoted by $=\sim$). Population values are included in the model via pre-multiplication of the right-hand side variables in each lavaan statement. Eta1 is regressed on (denoted by a single tilde $\sim$) Xi1, Xi2, and the interaction term denoted by Xi1:Xi2. All (residual) variances and covariances within the model (latent regression residuals, latent variances and covariances, or measurement errors) need to be stated ([co]variances are denoted by $\sim \sim$). The choice of the (residual) variances and covariances within the model will influence the proportion of explained variance within the structural model and the reliability. Both influence the power to identify an effect.

#### Using the powerNLSEM function for the estimation of power

After loading the powerNLSEM package and defining the model, we need to pass the model to the powerNLSEM function along with all hyper parameters that describe which model parameters we are interested in, which method to use to fit the QISEM, what power modeling method to use (probit, Wald, or logit) and which search method to apply. Further, we need to specify how many replications are to be performed what Type I error rate to use in the significance decisions or the prediction of power, and which power level should be achieved.

The first argument of the function is the model in lavaan syntax (model = ModerationModel). The POI are passed to the powerNLSEM function as a vector of strings that describe the parameters in lavaan syntax (POI = c("Eta1 $\sim$ Xi1:Xi2"), spaces in the syntax are ignored). If all regression parameters were of interest, this would result in POI = c("Eta1$\sim$Xi1", "Eta1$\sim$Xi2","Eta1$\sim$Xi1:Xi2"). However, the linear effects in a QISEM depend on the mean of the variables and are only comparable for zero mean variables (for a discussion of variable transformation problems in interaction models, see Moosbrugger et al., 1997). For an example of several POIs, see Fig. 8 in Appendix B.

For our example we use LMS (Klein & Moosbrugger, 2000) to fit the QISEM (method = "LMS"). We use probit regression to model power (power_modeling_method = "probit"), select a one-sided test (test = "onesided"), and use the *adaptive* algorithm (search_method = "ad- aptive") as we do not posses any prior knowledge on how large the required sample size is. Per default 10 steps are used, with increasing $R_j$, constrained relative change, a lower bound for sample size of $n_\text {lb}=5\cdot \# par$, as suggested by Wolf et al. (2013), a starting sample size of $n_\text {start}=10\cdot \# par$, where $\# par$ is the number of parameters within the model; all these arguments can be changed via the steps, distRj, constrainRelChange, nlb, and the N_start argument. The search is optimized for the required sample size to yield a power of 0.8 (power_aim = .8). In total, 2000 models (R = 2000) are fit. A Type I error rate of .05 is used for both the significance decision as well as the power estimation (alpha = .05, alpha_power_modeling = .05). 6 CPU cores are used (CORES = 6). For reproducability, a seed can be used (here, seed = 2024). # default indicates which of these options are default.

**Fig. 2:**
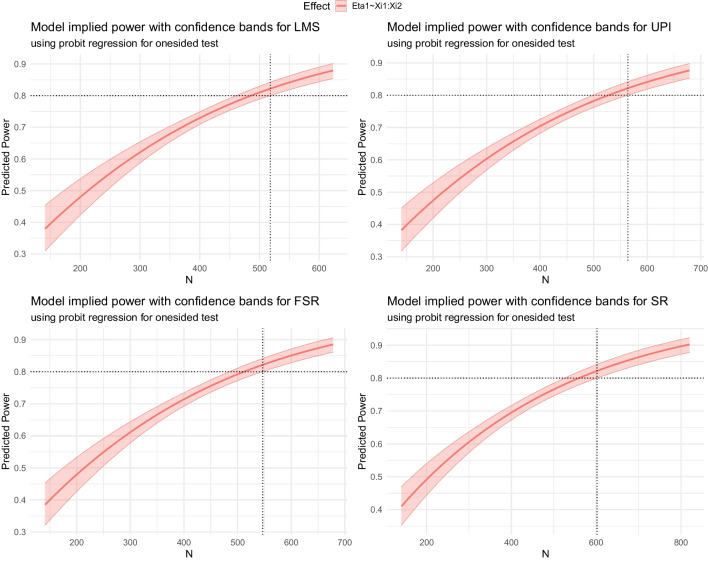
Predicted Power Rates for the Simple Latent Moderation Model Using LMS, UPI, FSR, and SR. *Note.* Output of plot(LMS_adaptive, se = TRUE), plot(UPI_adaptive, se = TRUE), plot(FSR_adaptive, se = TRUE), and plot(SR_adaptive, se = TRUE) with shaded area representing 95% confidence. The vertical dotted line represents the required sample size to yield a power rate of .8, indicated by the horizontal dotted line. The estimate for the required sample size using $R=2000$ replications within the MSPE were 518, 564, 547, and 602, respectively. For full code see Appendix D

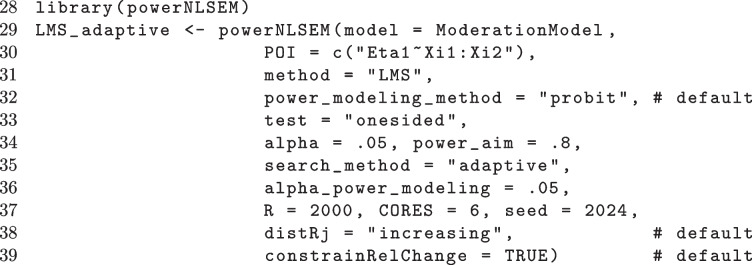



We note that the choice of the power level (via power _aim) is not irreversible. The power model can be reanalyzed after fitting using the reanalyse.powerNLSEM function, in which the resulting probit regression can be used to predict power and to solve for the required sample size. However, the power_aim argument does influence the choice of sample sizes used in the optimization process of the adaptive algorithm, but will not influence the required sample size other than via the stability (i.e., if the power rate of interest differs strongly from .8, then the required sample sizes can be less precise, see also simulation studies below).

Fitting took $\sim$30:48min on a standard computer^1^ using 6 cores. Runtime is influenced by the number of replications (R), the estimation method (method), the number of steps to take in the adaptive algorithm (steps), the sample sizes needed to yield the desired power rate, and, of course, the number of cores used on the computer. Very low sample sizes (less than 100) can result in difficulties in convergence and sample sizes well beyond 1000 can increase runtime. As the required sample size is influenced by the effect size, the power rate, and indirectly via the indicator reliability, coefficients in the model argument (model) as well as the power level (power_aim) influence runtime.

The LMS_adaptive object, and therefore the output of the powerNLSEM function is a list containing, among further information, the required sample size $\hat{N}_{\alpha , \text {lb}}$, as well as the parameter estimates and standard errors, from which the significance decision can be computed, performance evaluations on the average sample size weighted bias and relative bias, and a weighted RMSE which can be used to evaluate the performance of parameter estimation. See documentation of the powerNLSEM package (Irmer, 2024) for more details. -

**Table 1:** Results of the MSPE for LMS, UPI, FSR, and SR for a Single Trial

Method	$N_\alpha$	$\hat{N}_\alpha$	$\hat{N}_{\alpha ,\text {lb}}$	wBias	wRel-Bias	rwMSE	Convergence	Runtime
LMS	485	486	518	-0.0008	-0.83	0.0433	1.000	11,085
UPI	522	528	564	-0.0012	-1.25	0.0447	0.992	509
FSR	494	512	547	0.0020	1.99	0.0458	1.000	1,136
SR	542	560	602	-0.0090	-8.99	0.0417	1.000	415

*Note.* Result of MISE for LMS, UPI, FSR, and SR for single trial with seed = 2024 for $\rho =0.8$, wBias = sample size weighted parameter bias in estimation of QISEM, wRel-Bias = weighted relative parameter bias in estimation of QISEM in %, rwMSE = root weighted mean squared error of parameters in estimation of QISEM, selected sample sizes were used as weights, Convergence = convergence rate of fitted QISEM within MSPE, Runtime = total runtime in seconds, for actual runtime on machine divide by the number of cores (here = 6), R = 2000, $N_\alpha$ is based on $8\times 10^5$ replications using a probit regression model

#### The Use of alternative estimation methods

The estimation method for QISEM can easily be altered in the powerNLSEM function by the use of the method argument. Here, code line 31 needs to be changed to method = "UPI", method = "FSR", or method = "SR". This alters the estimation method from LMS to UPI, FSR, or SR, respectively. Per default, matched product indicators with double mean centering are used for the UPI approach and the SL method (named after Skrondal & Laake, 2001, as suggested by Ng & Chan, 2020) is used for FSR. Full code is given in Appendix D or the OSF repository https://osf.io/k79hv.

#### Visualization and performance evaluation

The results are visualized using the plot function in R, which returns a ggplot2 object (Wickham, 2016). Using the argument se = TRUE plots confidence bands around the model implied power rates (see Fig. 2).
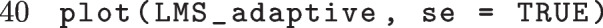


The lower bound of the confidence band equals the desired power rate for which the required sample size is computed. For this model and seed, the estimate for the required sample size using 2000 replications was 518 using LMS to fit the QISEM. For UPI, FSR, and SR, the required sample size resulted as $\hat{N}_{\alpha ,\text {lb}}$ = 564, 547, and 602, respectively. Table 1 shows $\hat{N}_{\alpha ,\text {lb}}$ and $\hat{N}_\alpha$ per method and the comparable $N_\alpha$ based on a probit model using $8 \times 10^5$ replications of the following simulation study. As expected, the ML approach, LMS, has the lowest required sample size as rather high reliabilities and a rather low model complexity was used. SR shows the largest required sample size. This could be due to the much lower reliability of the product terms in scale regressions compared to the main effects of $\xi _1$ and $\xi _2$ (see, e.g., Bohrnstedt & Marwell, 1978) which affects parameter estimation in regression with measurement error (Busemeyer & Jones, 1983, see also Dimitruk et al., 2007).

Finally, the summary function can be applied to the LMS_adaptive object to get further performance criteria such as the weighted bias, or weighted RMSE for parameter estimates of the *R* replications. These are further depicted in Table 1.
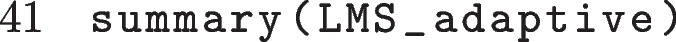


As expected SR showed the largest weighted (relative) bias with an average underestimation of $\sim -9\%$. The other methods showed small bias $<2\%$. The selected sample sizes were used as weights. The MSPE for LMS showed a runtime that was at least 10 times longer compared to all other methods. However, LMS had the smallest required sample size to yield a power of .8.

## The selection of N within MSPE analysis

In the following section, we use simulation to analyze the adaptive algorithm in comparison to the ad-hoc selection of sample sizes either with artificial significance decisions or within the powerNLSEM package. All code is provided at the OSF repository under https://osf.io/k79hv.

In this section we justify the selected (hyper-)parameters of the adaptive algorithm which we derive from a comparison to the best performing sample size constellation of ad-hoc selections of sample sizes. In their numerical illustration, Irmer et al. (2024) demonstrated that the use of the MSPE for power prediction and required sample size worked well with regard to bias, RMSE, and Type I error rates. However, they noted that the sample sizes used within the fitting of the probit regression might influence the precision. Further, the selected power rate interest $\rho$ influences the precision of the required sample size $N_\alpha$, with power rates $\rho \ge .99$ requiring large replication counts *R*. This is due to the nonlinear association between $\rho$ and $N_\alpha$. As a consequence, Irmer et al. (2024) used sample sizes that resulted in power rates smaller or equal to .99 when evaluating the MSPE as they limited replication counts to $R\le 2200$.

When conducting power analysis, determining the appropriate sample size can be challenging since the exact value is often unknown, and the expected interval for this size is uncertain. Nonetheless, sample sizes must be selected for simulation procedures. Hence, in order to use the MSPE in applied research, this would require prior knowledge about the size of the power rate per sample size to enable an educated choice of sample sizes within the simulation. In the following we examine the influence of the selection of sample size in two simulation studies, either with an ad-hoc selection of sample sizes in a given interval, denoted as the *brute-force* approach, and we propose an adaptive algorithm that automatically selects sample sizes, denoted as the *adaptive* approach. Both algorithms are implemented in the powerNLSEM package (Irmer, 2024) designed to estimate power for NLSEM.

As the MSPE using a probit regression model yields unbiased estimates for power and required sample size, two simulation studies were conducted using artificial MPSE data, where simulated significance decision were used, which resulted directly from a probit regression model with $\beta _0=-1.64$ and $\beta _1=.1$ or .2, which correspond to $N_\alpha = 616$ and 154, respectively. We did not simulate significance decision within the QISEM due to runtime constraints as the aim of the study was a detailed examination with high precision using $10^5$ replications per condition. Detailed description of the conditions is given next. For the simulation, 35 parallel processes on a shared computer cluster^2^ were used. The runtime was $\sim$5.5 days, which corresponds to $\sim$4609h total computation time. Using actual data within QISEM would increase runtime by a factor of $\ge 10^3$, which is why artificial significance decisions simulated from a probit regression model with root-*n* as a predictor were used.

We evaluated the performance of the two MSPE appro-aches by computing the bias, relative bias, the root mean squared error (RMSE) and the Type I error for the lower bound estimate for the sample size $\hat{N}_{\alpha ,\text {lb}}$. Therefore, bias cannot be expected to be zero, but bias should approach zero (from above) for large replication counts *R*. Hence, the conditions with the lowest bias (from above zero) and RMSE and Type I error rates closest to $2.5\%$ are the preferred constellations. Relative bias is computed by dividing the bias by $N_\alpha$.

$$\begin{aligned} \text {Bias} =&\frac{1}{R}\sum _{i=1}^R \hat{N}_{\alpha ,\text {lb},i}-N_\alpha , \, \text {RMSE} = \sqrt{\frac{1}{R}\sum _{i=1}^R \left( \hat{N}_{\alpha ,\text {lb},i}-N_\alpha \right) ^2}, \, \\&\text {Type I error} = \frac{1}{R}\sum _{i=1}^R \textbf{1}_{\{\hat{N}_{\alpha ,\text {lb},i}<N_\alpha \}}. \end{aligned}$$

### The brute-force approach to the selection of N

First the *model*, the (QISEM-)*method*, the true parameter values $\vartheta _0$, the parameters of interest *POI*, the desired power rate $\rho$ and the Type I error rate for significance decision $\alpha$ are selected, and a Type I error rate for the confidence band around $\rho$, $\alpha _\rho$, is set. The *brute-force* algorithm then uses the predefined sample sizes by the user by specifying a lower $n_\text {min}$ and an upper bound $n_\text {max}$ for the sample size and then drawing sample sizes either systematically or randomly from the interval $[n_\text {min}, n_\text {max}]$. The MSPE can then be used to interpolate the power beyond the user-defined interval of sample sizes. For better precision, a larger number of sample sizes can be selected (Irmer et al., 2024). The primary limitation of the brute-force approach is that the required sample size might not fall within the user-defined interval, i.e., $N_\alpha \notin [n_\text {min}, n_\text {max}]$. In such cases, the accuracy of the interpolation decreases as the distance from the fitted data increases. Pseudo code for the brute-force algorithm is given in Algorithm 2.

**Algorithm 2:**
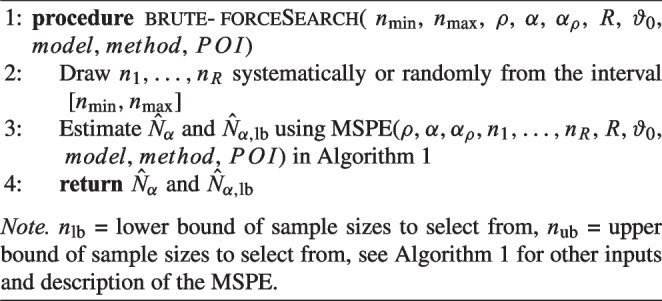
Pseudo code for the *brute-force* algorithm used in the powerNLSEM package.

To examine the performance of the brute-force approach, we conducted a simulation study in which we manipulated $n_\text {min}$ (25, 50, 100, 150, 200), $n_\text {max}$ (50, 100, 200, 300, 400, 500, 600), *R* ($10^3$, $10^4$) and $\beta _1$ (0.1, 0.2). Removing combination where $n_\text {min}\ge n_\text {max}$ resulted in a total of 140 conditions. For each condition we simulated $10^5$ replications of the MSPE with artificial significance decisions (simulated from a probit regression model with $\beta _0=-1.64$ and $\beta _1$ varying). Bias, root mean squared error (RMSE) and Type I error rates are displayed in Fig. 9 in Appendix B.

The results suggest that smaller $n_\text {min}$ resulted in overall higher performance, while for $n_\text {max}$ a minimum size was required for the performance to be acceptable. For $\beta _1=0.1$ this was the highest sample size used, i.e., performance was best for $n_\text {max}=600$, while for $\beta _1=0.2$ best performance was achieved for $n_\text {max}=300$ and performance slightly decreased for larger $n_\text {max}$. For larger $n_\text {max}$ for $\beta _1=0.2$ Bias and RMSE increased. This suggests that there is an optimal interval for fitting MSPE and *simply using a large interval* does not yield best results.

**Fig. 3:**
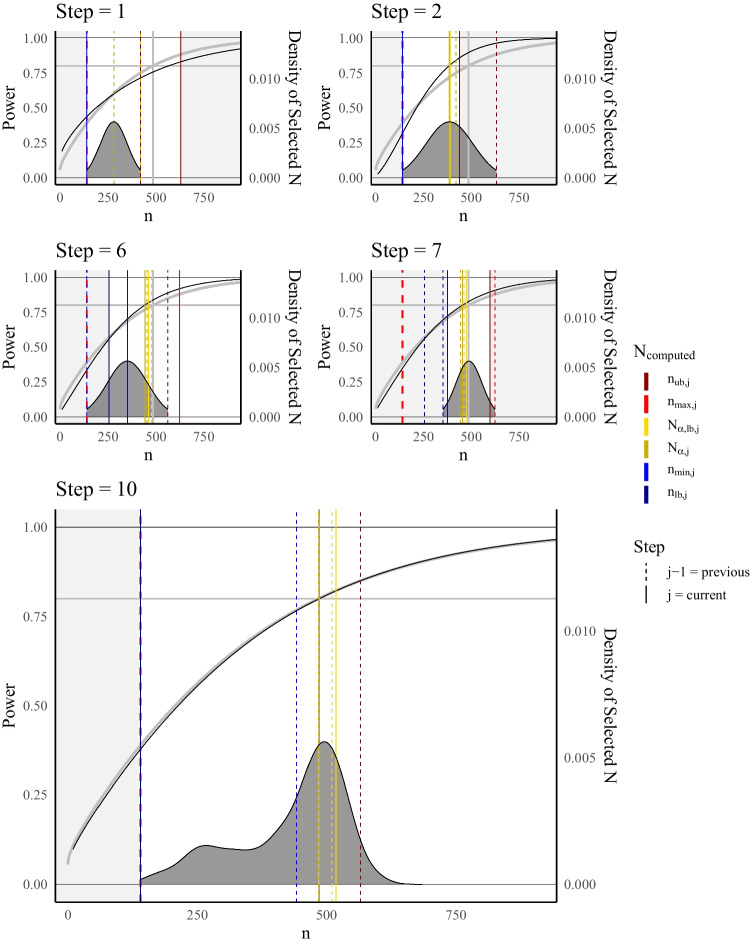
Illustration of the Adaptive Algorithm within MSPE for LMS. *Note.* Steps within the adaptive algorithm for selection of samples sizes withing MSPE. Bold grey curve = reference probit regression model based on $8\times 10^5$ (see Simulation Study 1), vertical bold grey line = reference required sample size $\tilde{N}_\alpha$, black curve = computed MSPE based on samples until given step, densities represent distribution of selected sample sizes based on current estimation of MSPE for step 1,2, 6, and 7, while for step 10, the actually used sample size distribution is shown with the right vertical axis corresponding to the empirical density. Bold dashed lined = lower bound of possible sample sizes $n_\text {lb}$, color = computed sample sizes based on step *j* and $j-1$, line type = previous and current step. For pseudo code and further explanation see Algorithm 3

### The adaptive approach to the selection of N

The results of the previous section suggest that there is an optimal interval of sample sizes used within MSPE. This is why we propose the adaptive algorithm that dynamically adjusts the selected sample sizes to yield optimal predictions of power. A slightly simplified version of the algorithm is depicted in Fig. 3 and detailed pseudo code is given in Algorithm 3.

The *adaptive* algorithm has a predefined number of *steps*. First, a starting sample size $n_\text {start}$ is selected, which should be set proportional to the complexity of the model and the number of variables, in order to result in reasonable convergence rates (for minimum sample size requirements in SEM, see e.g., Wolf et al., 2013). The MSPE is then fitted to a subset of size $R_1$ of the total *R* samples distributed symmetrically around $n_\text {start}$ in the interval $[n_{\text {min},1}, n_{\text {max},1}]$, where $n_{\text {min},1} = n_{\text {start}}/2$ and $n_{\text {max},1} = 2n_{\text {start}}$. To optimize the differentiation between significant and non-significant estimates in the QISEM, we focus on estimated powers around .5 in a broad search, where the power curve exhibits its steepest slope, i.e., highest first derivative. Sample sizes near this estimated power of .5 contain the most statistical information about the model concerning the relationship between power an sample size. We use the result from the MSPE to estimate the sample size that would yield a power of .15, .5,  and .85, $n_{\rho =0.15}, n_{\rho =0.5}$, and $n_{\rho =0.85}$, respectively. To ensure more stability in the interval tails, new drawn sample sizes are symmetrically distributed around the one that yields a power of .5 within an interval where the lower bound guarantees a power of at least .15, and the upper bound ensures a power of .85. Hence, for step $j\ge 2$, $R_j$ sample sizes are distributed symmetrically in the interval $[n_{\text {min},j}, n_{\text {max},j}]$, where an estimate for $n_{\text {min},j}$ is $n_{\rho =0.15,j}$ and an estimate for $n_{\text {max},1}$ is $n_{{\rho =0.85},j}$, where $\sum _{j=1}^{steps}R_j = R$. The newly drawn sample sizes are added to the existing ones and significance decision are only computed for the newly drawn sample sizes. All available sample sizes and significance decision are used within the MSPE in each step. This process is repeated several times to achieve stability in parameter estimation within the MPSE. After several repetitions (e.g., at $j\ge switchStep = steps/2$), the algorithm switches its focus from wide search to narrow search and starts selecting sample sizes to distribute around the target sample size $n_\rho$, which ensures a power of the desired level $\rho$, i.e., sample sizes are distributed symmetrically in the interval $[n_{\text {min},j}, n_{\text {max},j}]$ that includes $n_\rho$, in an narrow search phase. These sample sizes are computed by solving for a lower and an upper bound around $\rho$: $n_{\rho _\text {lb},j},$ and $n_{\rho _\text {ub},j}$ are computed in every step. Sample sizes that are too small result in faulty convergences or improper solutions (Wolf et al., 2013). Thus, when randomly selecting sample sizes, it is important to consider this aspect. A common lower bound in SEM analyses is $n_\text {lb}=5\cdot \#par$, where $\#par$ represents the number of parameters within the model (Wolf et al., 2013). Additionally, we manipulated the focus of the search by constraining the interval of sample sizes within each step. This is why within each step *j* the bounds of the selected interval are evaluated whether they fall outside a certain bound. A step dependent lower ($n_{\text {lb},j}$) and upper bound ($n_{\text {ub},j}$) are implied by the focus of search algorithm. Hence, it is checked whether the bounds for the interval of newly drawn samples sizes ($n_{\text {min},j},\ n_{\text {max},j}$) either fall below the lower bound of accepted sample sizes $\max \{n_\text {lb},\ n_{\text {lb},j}\}$, which should ensure acceptable convergence rates, or exceed a certain upper bound $n_{\text {ub},j}$. All bounds are allowed to change with *j* which ensures that the MSPE does not suffer from faulty convergence of the probit regression step or from the large standard errors in the initial steps. Consequently, within every step $n_{\text {min},j} = \max \{n_{\rho _\text {lb},j},\ n_{\text {lb},j},\ n_\text {lb}\}$ and $n_{\text {max},j} = \min \{n_{\rho _\text {ub},j},\ n_{\text {ub},j}\}$, with for $j=1$, $n_{\rho _\text {lb},1}=n_{\text {lb},1}=n_\text {start}/2$ and $n_{\rho _\text {ub},1}=n_{\text {ub},1}=2n_\text {start}$. The distribution (*distRj*) of the $R_j$ can further be manipulated to control the weighting of wide search and narrow search.

By transitioning from wide to narrow search and evaluating the sample sizes in several steps, the adaptive algorithm is flexible. Even if the initial sample size is far from the required one, the algorithm can adapt and fine-tune the search process to ensure it eventually finds the required sample size. This makes the adaptive algorithm a powerful tool for optimizing sample size selection in the MSPE process. Pseudo code is provided in Algorithm 3 and the algorithm is illustrated in Fig. 3.

**Algorithm 3:**
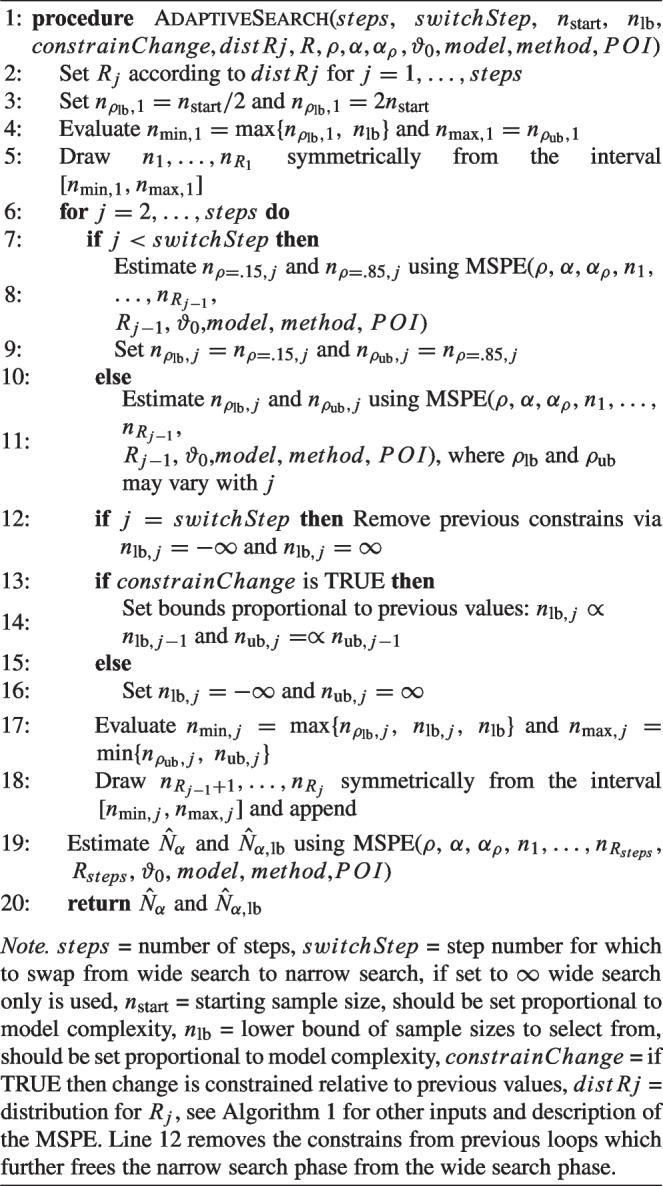
Pseudo code for the *adaptive* algorithm used in the powerNLSEM package.

The argument *constrainChange* controls whether the upper and lower bound of the newly drawn sample sizes should be evaluated and constrained proportional to previous values of the lower and upper bound. If the process should be unconstrained, then $n_{\text {lb},j}=-\infty$ and $n_{\text {ub},j}=\infty$.

To evaluate the performance of the adaptive approach, we conducted a simulation study in which we manipulated $n_\text {lb}$ (25, 50), $n_\text {start}$ (75, 150), the number of *steps* (2, 5, 10), the position where the algorithm should switch from wide search to narrow search ($switchStep=$ *step*/2 or $\infty$; i.e., wide search only), whether changes in predicted sample sizes should be constrained to a maximum relative change (constrained, free), the distribution of replications $R_j$ for $j=1,\dots ,steps$ (u-shaped, increasing, equal), *R* ($10^3$, $10^4$) and $\beta _1$ (0.1, 0.2). This resulted in a total of 576 conditions. For each condition we simulated $10^5$ replications of the MSPE with artificial significance decisions. Bias, root mean squared error (RMSE) and Type I error rates are displayed in Fig. 10 in Appendix B. The results indicate that bias decreased with increasing $n_\text {start}$, albeit resulting in larger initial intervals of sample sizes. Moreover, bias decreased with smaller $n_\text {lb}$ and larger *R*. Generally acceptable performance was observed only for constrained changes and 5 or 10 steps (see Fig. 10 in Appendix B).

**Fig. 4:**
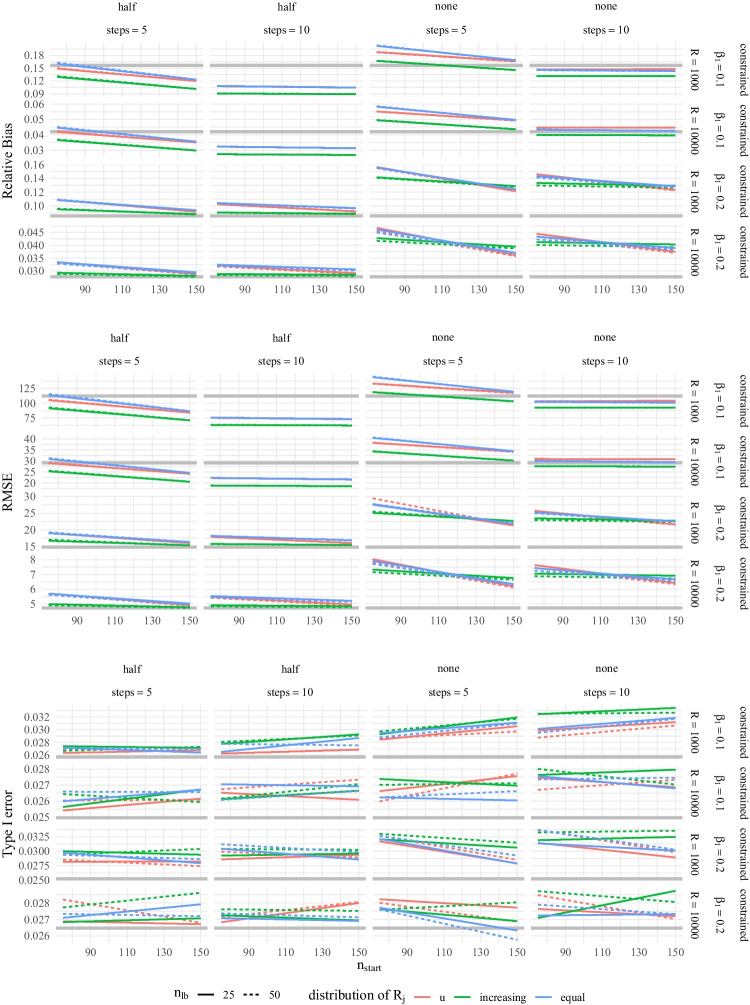
Relative Bias, RMSE and Type I Error Rate of the Best Performing Parameter Constellations of the Adaptive Algorithm in Comparison with the Best Performance of the Brute-force Algorithm within MSPE. *Note.* Relative bias, RMSE, and Type I error rate of the best performing parameter constellations of the adaptive algorithm in comparison with the best performance of the brute-force algorithm within MSPE (in gray) plotted against $n_\text {start}$ for different numbers of steps and switches (at half of the steps or none) in columns and differing $\beta _1$ and replication counts *R* in rows. Line types and color represent lower bound of search space $n_\text {lb}$ and distribution type of drawn sample sizes among steps *distRj* (u = u-shaped)

**Table 2:** Comparison in Bias, Relative Bias, RMSE, and Type I Error Rate for the Best Performing Adaptive Search and brute-force Constellations in MSPE

Condition		Bias		Relative bias		RMSE		Type I error	
$\beta _1$	R	Adaptive	Brute	Adaptive	Brute	Adaptive	Brute	Adaptive	Brute
0.1	1,000	54.94	96.53	0.089	0.157	62.37	112.29	0.029	0.026
0.1	10,000	16.53	25.94	0.027	0.042	18.53	29.25	0.027	0.025
0.2	1,000	13.54	13.15	0.088	0.085	15.37	14.77	0.030	0.025
0.2	10,000	4.32	4.28	0.028	0.028	4.77	4.72	0.028	0.026

*Note.* Bias, relative bias, and RMSE are computed using the lower bound estimate $\hat{N}_{\alpha ,\text {lb}}$ compared to the population sample size $N_\alpha$, $\beta _1$ = slope of probit regression, *R* = number of replications

The adaptive algorithm is expected to perform at least equally as well, if not better, than the brute-force algorithm, even under ideal circumstances with the best a-priori selected interval of sample sizes. To further assess this, we compared the promising configurations of the adaptive algorithm to the best performing condition of the brute-force algorithm in Fig. 4. The (relative) bias and RMSE decreased with increasing $n_\text {start}$ and *R*, while $n_\text {lb}$ exhibited only a minor influence. Best performance in terms of (relative) bias and RMSE was observed with increasing $R_j$. Adaptive algorithms with a *switchStep* set at half of the total steps (*steps*/2) showed better performance compared to no switches. For $\beta _1 = 0.1$, the adaptive algorithm outperformed the brute-force algorithm, although this was influenced by the choice of the search interval within the brute-force algorithm. Notably, the adaptive algorithm exhibited comparable relative bias for $\beta _1 = 0.1$ and 0.2, indicating its flexibility, while the brute-force algorithm showed a much larger relative bias for $\beta _1 = 0.1$. Using 10 steps resulted in slightly better performance compared to 5 steps. Type I error rates were slightly elevated but generally acceptable.

Overall, the most promising parameter constellation for the adaptive algorithm is as follows: 10 steps, with $switchStep$ at 5, $n_\text {start} = 150$, $n_\text {lb} = 50$, increasing $R_j$, and a constrained search. Table 2 shows the bias, relative bias, RMSE, and Type I error rate for the best performing parameter constellations for the adaptive and brute-force algorithm. The best parameter constellation for the adaptive algorithm is implemented in the powerNLSEM package (Irmer, 2024) as defaults.

## Simulation studies to examine the performance of the MSPE within the powerNLSEM package

In order to illustrate the performance of the MSPE method for QISEM, we conducted two simulation studies for a latent moderation model and a more complex moderated mediation model. The model formulation is given in Appendix A in lavaan syntax. The previous simulation study examined several constellations of (hyper-)parameters controlling the adaptive algorithm. In this section, we employ the best-performing constellation of (hyper-)parameters for the adaptive algorithm and compare it to the brute-force algorithm using the methods implemented in the powerNLSEM package. Instead of simulated significance decisions, we utilize real simulated data to examine the effect of model complexity and reliability on the performance of the adaptive algorithm within MSPE. Additionally, we compare the four estimation methods concerning required sample sizes.

### Simulation study 1: Power estimation in a simple latent moderation model

Simulation Study 1 replicates the power estimation procedure of the tutorial for the simple latent moderation model depicted in Fig. 1 in order to examine the distribution of the required sample sizes for different power levels for LMS, UPI, FSR, and SR. We manipulated the reliabilities (high vs. low) and examined the two search methods using $R=2000$ replications: adaptive search (the desired power level for optimization was .8) and the brute-force algorithm using $R_j=1000$ different sample sizes ($n_1=140,\dots ,$ $n_{1000}=1139$ with 2 replications each). The lower bound of the brute-force algorithm is identical to $n_\text {lb}=5\cdot \# par$ (see previous section) within the adaptive algorithm motivated by the lower bound in SEM (Wolf et al., 2013). We used the interaction effect as the POI.

For our simulation, we used 35 parallel processes on a shared computer cluster^3^. Since this simulation merely serves as a demonstration and the runtime was $\sim$2.3 days, which corresponds to $\sim$1922h total computation time, we limited Simulation Study 1 to 200 replications for each condition. As an estimation of the true required sample size per power level, we used all simulated significance decisions per reliability condition and estimation method ($8\times 10^5$ in total per condition and method) to estimate a more precise prediction for the required sample size for different power levels (.5, .6, .7, .8, .9,  .95) using a probit regression model. Irmer et al. (2024) showed that the approximation of power using the MSPE works well and that the procedure is unbiased. Hence, we used many replications as a proxy for the population values but acknowledge small sample variations.

**Fig. 5:**
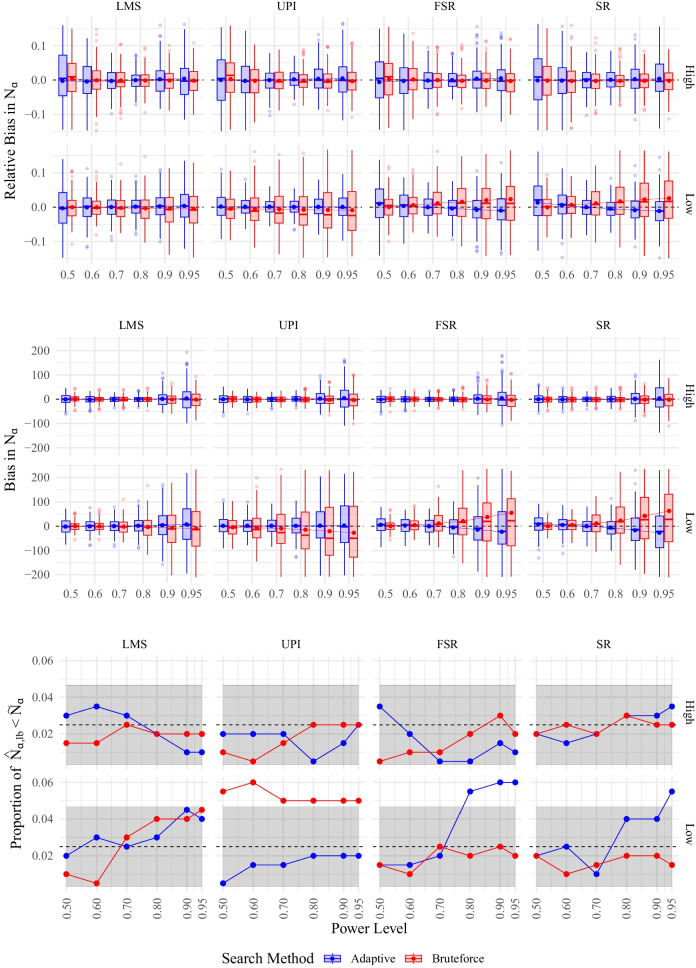
(Relative) Bias and Type I Error Rate of the MSPE for the Simple Moderation Model. *Note.* Relative bias, bias and Type I error rate for the MSPE using the adaptive and the brute-force algorithm for the LMS, UPI, FSR, and SR estimation methods for different power rates $\rho$ and reliabilites (high vs. low). For boxplots, 1% of extreme values were removed for clarity, shaded area represents confidence band around expected Type I error rate of 2.5%. Required sample size $\tilde{N}_\alpha$ was computed using all available information (i.e., all $\sim 8\times 10^5$ replications per estimation method). Dashed lines indicate preferred values

**Fig. 6:**
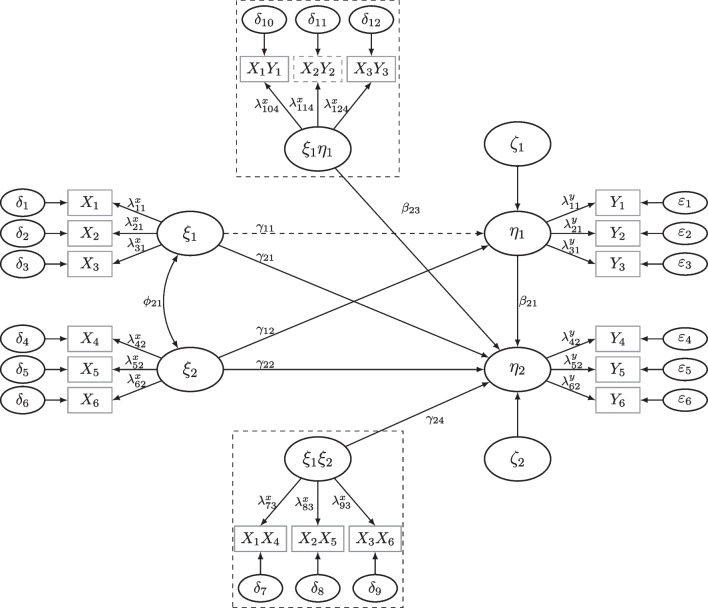
QISEM Including Moderation and Moderated Mediation Effects. *Note.* Mean structure and intercepts are not included in the figure. Correlation among nonlinear terms is not drawn for clarity. Dashed box shows product indicators formed within the unconstrained product indicator approach used to fit the QISEM

Figure 5 displays the results of the simulation study (Tables 3 and 4 in Appendix C list the numerical values). In the high reliability condition, both the brute-force and adaptive algorithms exhibited almost no bias. However, in the low reliability condition, the brute-force algorithm tended to overestimate the required sample size. In contrast, the adaptive algorithm maintained acceptable bias for power rates $\le 0.8$ across all methods. Bias was also deemed acceptable for LMS and UPI even for higher power rates. However, for power rates exceeding 0.8, FSR and SR showed slight underestimation of the required sample size. Further details can be found in Table 3 and 4 in Appendix C. Given that the adaptive algorithm targeted a power rate of 0.8, its bias remained acceptable for the desired power rate across all conditions. Additionally, almost all biases observed for both the adaptive and brute-force algorithms were within $10\%$ of the reference required sample size. Type I error rates generally remained within acceptable bounds, with slight elevations observed for large required power rates for FSR and SR under the low reliability condition for the adaptive algorithm. Moreover, Type I error rates were slightly elevated across all power rates for SR in the brute-force algorithm under the low reliability condition.

The estimation of required sample sizes exhibited the lowest bias for power rates of 0.8 across all methods for the adaptive algorithm. Computed required sample sizes were smallest for LMS, followed by FSR, UPI, and SR for the high reliability condition, while for the low reliability condition, LMS also had the smallest required sample sizes, followed by FSR, SR and UPI (see Table 3 and 4 in Appendix C). Hence, for low reliabilities, UPI requires the highest sample sizes to yield the desired power rates.

In summary, the adaptive algorithm of MSPE for LMS, UPI, FSR, and SR as estimation methods for QISEM demonstrated favorable performance regarding bias, relative bias, and Type I error rates. In contrast, the brute-force algorithm only displayed satisfactory performance when the interval of selected sample sizes encompassed the required sample size, replicating the simulation results comparing the adaptive and brute-force algorithms for artificial simulated data. Notably, much larger sample sizes are required for all estimation methods for QISEM under low reliabilities.

### Simulation study 2: Power estimation in a moderated mediation model

Since the distribution of required sample sizes of the brute-force approach to sample selection in MSPE was similar to that of the adaptive algorithm under the high reliability condition of the previous section, and resulted in overestimated sample sizes under the low reliability condition for all methods of QISEM, we limited Simulation Study 2 to the adaptive algorithm using probit regression to predict power. The desired power level for optimization was set to .8. We, again, compared the performance for LMS, UPI, FSR, and SR on fitting a moderated mediation model with two moderation effects. The structural model of the resulting QISEM can be represented as follow (see Fig. 6)

$$\begin{aligned} \eta _1&= .2\xi _1 + .5\xi _2 + \zeta _1,\\ \eta _2&= .2\eta _1 + .1\eta _1\xi _1 + .2\xi _1 + .3\xi _2 + .1\xi _1\xi _2 + \zeta _2, \end{aligned}$$

with all variables having a variance of 1, hence $\mathbb {V}ar \, \zeta _1=.61$ and $\mathbb {V}ar \, \zeta _2=.620975$, and a correlation between the standardized predictor variables $\xi _1$ and $\xi _2$ of .5. Some computations were performed using Maple (Maplesoft, a division of Waterloo Maple Inc., 2019). We selected the two moderation effects as the POI: $\eta _1\xi _1\rightarrow \eta _2$ and $\xi _1\xi _2\rightarrow \eta _2$.

Within this model, $\xi _1$ can be interpreted as the moderator variable moderating both the effect of $\xi _2$ on $\eta _2$ ($\xi _2\rightarrow \eta _2$) as well as the effect of $\eta _1$ on $\eta _2$ ($\eta _1\rightarrow \eta _2$). Further, the mediation of $\xi _2$ to $\eta _2$ ($\xi _2\rightarrow \eta _1\rightarrow \eta _2$) is therefore moderated. The moderation effects were set to be .1, which translates to a variance increment of $1.25\%$ for the effect of $\xi _1\xi _2\rightarrow \eta _2$ and $2.85\%$ of $\xi _1\eta _1\rightarrow \eta _2$ (for further information see Appendix A). Such a model is common in applied research, with some models including even more moderation effects (see, e.g., Ajzen & Kruglanski, 2019).

Compared to Simulation Study 1, we only considered the high reliability condition in Simulation Study 2. However, we altered the number of replications for UPI, FSR, and SR, as a new condition to examine the precision of the MSPE which is relevant for an actual application. We could not increase *R* for LMS due to time constraints. In total we simulated 100 replications of the adaptive search algorithm based on $R=2000$ samples for all methods and based on $R=10^5$ samples for UPI, FSR, and SR within the MSPE for QISEM. We, again, compared the performance of the MSPE to estimate required sample sizes using a probit regression model utilizing all significance decisions per method ($2\times 10^5$ in total for LMS; $102\times 10^5$ in total for UPI, FSR, and SR, see previous section for further descriptions). We used 35 processes on the shared computer cluster of the previous section, which took $\sim$ 13.1 days, which corresponds to $\sim$ 11011 h total computation time. This is why we limited Simulation Study 2 to 100 replications for each condition.

To translate the computation time from the server to an actual application, we timed each approach for a single trial on standard machine using 6 parallel processes. For $R=2000$, computation time was comparable for UPI, FSR, and SR to the simple QISEM, while for LMS the computation time was $\sim 21h$. For $10^5$, the computation time on 6 parallel processes was $\sim 48 min$ for UPI, $\sim 92 min$ for FSR, and $\sim 34 min$ for SR. For LMS an estimated computation time of $\sim 43.9 d$ would result.

**Fig. 7:**
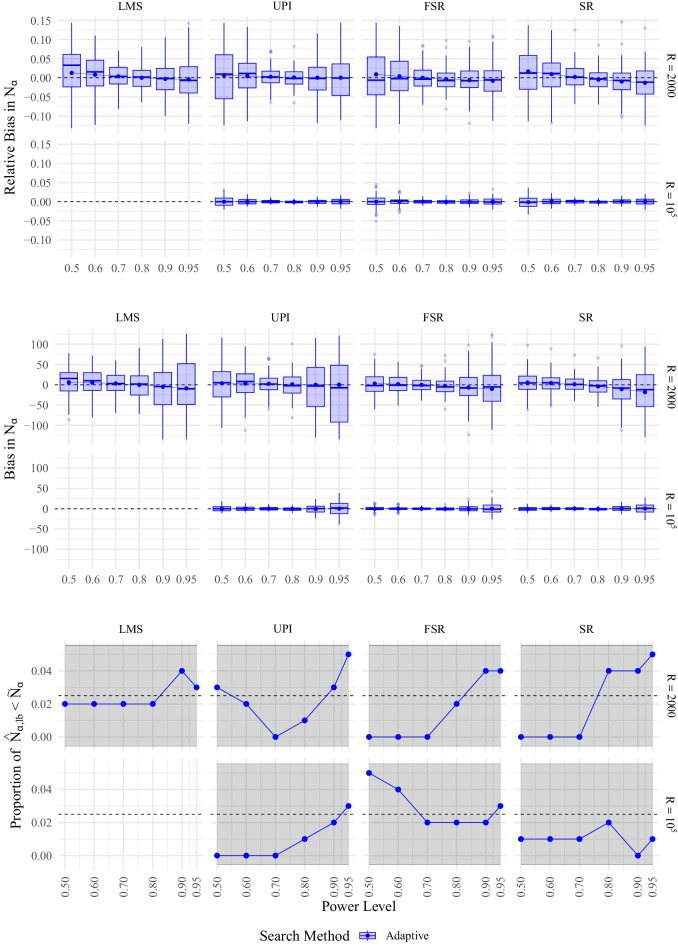
(Relative) Bias, and Type I Error Rate of the MSPE for the Complex QISEM. *Note.* Relative bias, bias and Type I error rate for the MSPE using the adaptive algorithm to model power for the LMS, UPI, FSR, and SR estimation methods for different power rates $\rho$ for the complex QISEM. For boxplots, 1% of extreme values were removed for clarity, shaded area represents confidence band around expected Type I error rate of 2.5%. Reference required sample size $\tilde{N}_\alpha$ was computed using all available information (i.e., all $\sim 2\times 10^5$ replications for LMS and $102\times 10^5$ for UPI, FSR, and SR). Rows indicate different replication counts $R=2000$ and $R=10^5$. Dashed lines indicate preferred values. LMS was not used in the $R=10^5$ condition due to the long runtime

**Fig. 8:**
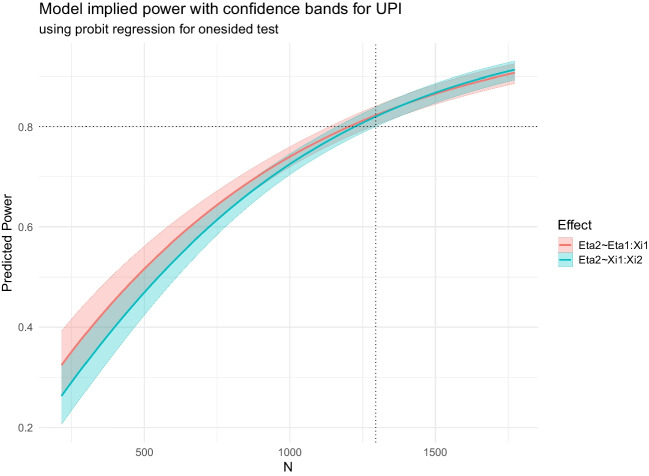
Predicted Power Using the Adaptive Algorithm within MSPE for UPI in the Complex Model. *Note*. Output of plot for the adaptive algorithm within MSPE for UPI for $R=2000$ replications for the complex QISEM. Runtime was  1.4min. Shaded areas are 95% confidence bands for the probit regression model within MSPE. Vertical line represents the required sample size $\hat{N}_{\alpha ,\text {lb}}=1295$, horizontal line represents the requested power rate $\rho =.8$

**Fig. 9:**
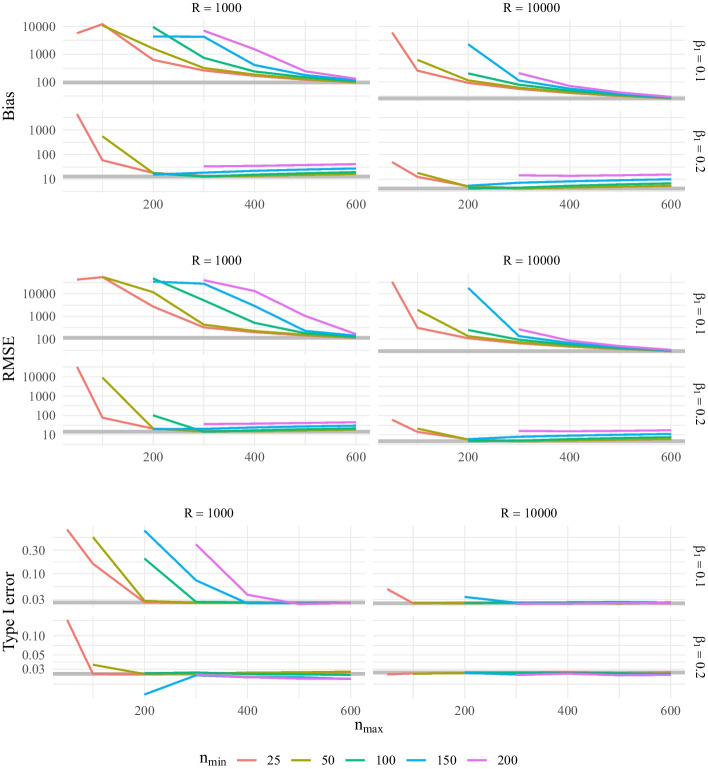
Bias, RMSE and Type I Error Rate of the Parameter Constellations of the Brute-Force Algorithm. *Note*. Bias, RMSE and Type I error rate of the parameter constellations of the brute-force algorithm in comparison with the best performance of the brute-force algorithm (in gray) for different replication counts *R* in columns, and differing $\beta _1$ in rows vs $n_\text {max}$. Line colors represent $n_\text {min}$

**Fig. 10:**
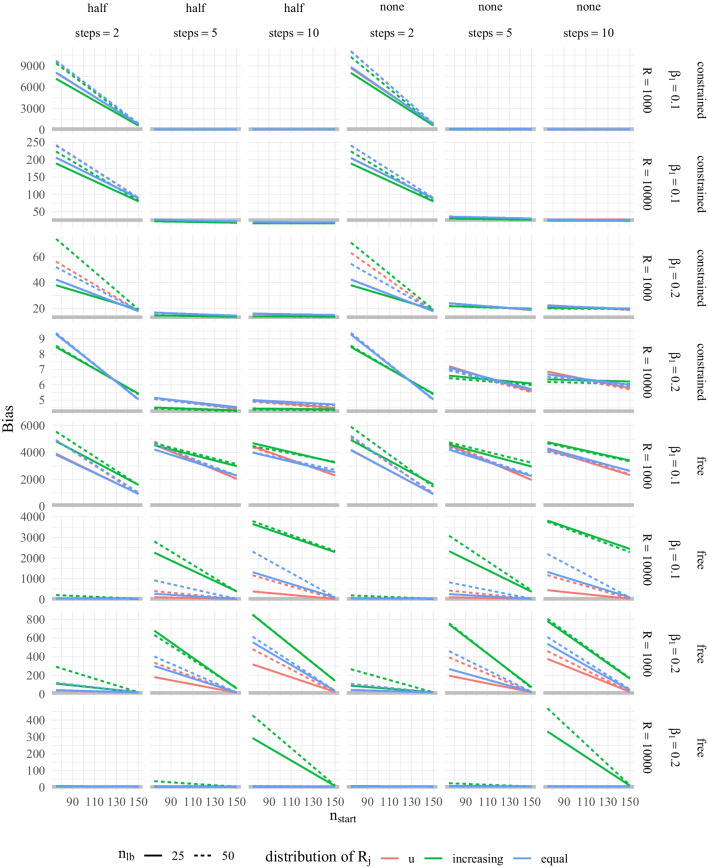
Bias of All Parameter Constellations of the Adaptive Algorithm within the MSPE. *Note*. Bias of the parameter constellations of the adaptive algorithm within the MSPE for different numbers of steps and switches (at half of the steps or none) in columns, and differing change constrains (constrained vs. free) $\beta _1$ and replication counts *R* in rows. Line types and color represent lower bound of search space $n_\text {lb}$ and distribution type of drawn sample sizes among steps *distRj* (u = u-shaped)

The moderation effect of $\xi _1$ on the effect of $\eta _1\rightarrow \eta _2$ ($\xi _1\eta _1\rightarrow \eta _2$) had the smallest power for each sample size and method, and, therefore, was the decisive influence for the required sample size, hence, the required sample size is only influenced by the power of this effect. Figure 7 depicts bias and relative bias as well as Type I error rates for the MSPE to model power for the four methods to estimate QISEM (see also Table 5 and 6 in Appendix C for numerical value). Required sample sizes were higher compared to the high reliability condition of Simulation Study 1. This is most probably due to the higher model complexity. Bias was smallest for the required sample size resulting from a power rate of .8 which came of no surprise as this was the power rate the adaptive algorithm was optimizing for. Required sample sizes were slightly overestimated for power rates smaller than .8 and slightly underestimated for power rates larger than .8. However, relative bias in required sample sizes per replication were almost all smaller than 10% and average relative bias across replications was almost 0. Bias and relative bias was negligible for the $R=10^5$ conditon. All Type I error rates fell in the accepted interval.

Higher replication counts resulted in higher precision. For instance, the standard deviation and range in $\hat{N}_\alpha$ for a power rate of .8 were at least 668 % and 776% larger, respectively, for $R=2000$ compared to $R=10^5$ (see also Table 5 and 6 in Appendix C). The maximum absolute difference between the mean required sample size $N_\alpha$ and the required corresponding to the lower bound of the confidence band around the power rate $N_{\alpha ,\text {lb}}$ to yield a power of .8 was 3.4 highlighting the high precision. Similarly to Simulation Study 1, this indicates that larger replication counts are necessary to gain high precision, especially if high power rates are of interest. In contrast to the results of Simulation Study 1, the results of Simulation Study 2 revealed smaller required sample sizes for SR compared to LMS.

To summarize, required sample sizes for the moderation effects in the more complex QISEM were smaller for SR compared to LMS with the adaptive algorithm resulting in good approximations for required sample sizes. LMS requiring higher sample sizes for a regression coefficient compared to SR indicates the unique requirements per model amplifying the necessity for power estimation in such models as rule of thumb sample sizes cannot be generalized from similar models. Overall, the adaptive algorithm showed good performance for LMS, UPI, FSR, and SR with regard to bias and Type I error rates for this particular model. However, we note that for larger power rates more replications *R* are necessary to yield a higher precision in the estimation of the required sample size. Computation time for large replication counts is acceptable for UPI, FSR, and SR.

## Discussion

In this paper, we provided a tutorial to conduct power estimation for nonlinear SEM using the model-implied simulation-based power estimation (MSPE) as proposed by Irmer et al. (2024) in R (R Core Team, 2023) using the powerNLSEM (Irmer, 2024) package. The MSPE utilizes the distribution of parameter estimates and models their significance decision by a probit regression model with the square-root of the sample size as a predictor (Irmer et al., 2024). By the use of a parametric model, the MSPE is based on all correlation information between power rate and sample size and, hence, is an efficient method to describe the power curve. As there is no analytical solution for power analysis for nonlinear SEM, we proposed to use this MSPE method to estimate power in models that include quadratic and interaction/moderation effects, i.e., QISEM, fitted with latent moderated structural equations (LMS, Klein & Moosbrugger, 2000), the unconstrained product indicator (UPI, Kelava & Brandt, 2009; Marsh et al., 2004) approach, a simple factor score regression (FSR) approach to QISEM (as proposed by Ng & Chan, 2020) or the single indicator scale regression (SR) approach (scale means used a proxy per latent variable in a path model).

A challenge within MSPE was determining suitable sample sizes for the probit regression process. To address this, we proposed an adaptive algorithm. The adaptive algorithm dynamically adjusts sample sizes during the fitting process. Even when the initial sample size deviates significantly from the required one, the algorithm can dynamically adjust and optimize the search process, ensuring eventual attainment of the target sample size. This adaptability renders the adaptive algorithm a potent tool for optimizing sample size selection within the context of the MSPE method. As Irmer et al. (2024) showed via simulation and derivation that significance decisions in QISEM follow a probit regression model with square root of sample size as a predictor, we used artifical significance decisions within an extensive simulation study to compare the adaptive algorithm to the brute-force algorithm using a-priori selected sample sizes. The comparison was used to select the best (hyper-)parameter constellations for the adaptive adaptive algorithm. These were then used within the adaptive algorithm to select samples sizes within MSPE in the simulation studies examining power estimation in QISEM and were implemented in the powerNLSEM (Irmer, 2024) package.

We offered a comprehensive guide on employing the MSPE approach for QISEM with LMS, UPI, FSR, or SR using the powerNLSEM (Irmer, 2024) R-package, specifically leveraging the adaptive algorithm. We illustrated the performance of the adaptive algorithm compared to the brute-force algorithm for a simple moderation model for the LMS, UPI, FSR, and SR approach. Notably, the adaptive search algorithm demonstrated enhanced flexibility in various scenarios as the user does not need to provide any sample sizes before usage. However, the brute-force approach showed good performance as long as the required sample size fell within the interval of selected sample sizes, hence, if prior knowledge on the sample size of interest is available, the brute-force approach is useful. This was in line with the results of the extensive simulation study examining the artificial significance decisions.

Interestingly, we found that LMS required smaller sample sizes to achieve equivalent power rates compared to UPI, FSR, and SR, when analyzing the nonlinear effects in the smaller latent moderation model but found the opposite trend in the more complex QISEM. The first finding contrasts with prior research on linear SEMs, where single indicator approaches, such as the FSR and SR approach, exhibited higher power rates compared to techniques modeling overall measurement unreliability, such as linear SEM (see, e.g., Savalei, 2019). Therefore, this result emphasizes the unique relation between power and sample size for each coefficient for every model. Further, the results suggest that next to the effect size, reliability and model complexity influence the required sample size for the methods. This makes model-specific power estimation necessary and no rule of thumb for power estimation should be used. Finally, we conducted a simulation study examining the performance of the MSPE for a more complex moderated mediation model with two interaction effects. Overall, performance was good with regard to bias, relative bias, and Type I error rates, but runtime was especially large for LMS. UPI, FSR, and SR showed acceptable runtimes enabling the applied researcher to use large replication counts to increase precision.

Hence, the use of MSPE in combination with the adaptive approach in selecting sample sizes to conduct power analysis enables researcher to plan their research designs as well as examine non-significant results by conducting power analyses for smallest effect sizes of interest (Anvari & Lakens, 2021). Precise estimates of required sample sizes were only possible with very large replication counts (e.g., $R=10^5$).

However, the new method does not come without drawbacks. In the following, we discuss limitations and future research for the MSPE method and the powerNLSEM package, and we give practical recommendations when performing MSPE using the powerNLSEM package.

### Limitations and future research

Within mediation analysis, several approaches exist to test the mediation or indirect effects (Preacher & Hayes, 2008). Sobel (1986, 1982) proposed to use the Delta-method to derive standard errors for the product of the effects involved in the mediation effect and to utilize the *z*-test to evaluate the significance of the indirect effect. However, the Delta-method is an asymptotic procedure that requires large sample sizes. For smaller samples, bootstrapping methods were suggested to be used instead (Preacher & Hayes, 2008) as the indirect effect is not normally distributed in small samples. Hence, it is an open question whether the MSPE is applicable for testing functions of parameters such as the indirect effect in mediation analysis. If this is the case, the analysis could be extended to moderated mediation analysis to analyze conditional indirect effects (as described in Preacher et al., 2007).

The LMS methods requires extensively long runtimes for complex models, resulting in immense computational burden for the MSPE using LMS to fit QISEM. However, since the required sample size for LMS differs from that of the UPI, FSR, or SR method for different model complexities in different directions, it is not generally advisable to use the required sample size derived by another method instead, and this should only be done with caution. Future research should address the systematics of the order of required sample sizes for the methods.

By means of our simulation study examining the adaptive adaptive algorithm to the selection of sample sizes within MSPE, we were only able to examine a limited number of combinations of parameters, hence, the choice of defaults in the powerNLSEM package are based on limited information on parameters influencing the performance of the adaptive algorithm in MSPE.

The QISEM analyzed in the reported studies are rather simple and can be extended for instance for hierarchical data structures. Future research should examine whether the MSPE is applicable and practical to use for hierarchical SEM with interaction and random effects.

Finally, future research should examine whether the MSPE can be extended for Bayesian analyses. This is an important matter, since many complex SEM are fitted using the Bayesian framework.

### Features not included in the powerNLSEM package

The MSPE method for QISEM within the powerNLSEM package is only applicable for the LMS, UPI, FSR, or the SR method. For the LMS method to be usable, an implementation of M*plus* (Muthén & Muthén, 1998-2017) is necessary. However, as the MSPE approach can be used with almost any estimator that is asymptotically normal, further methods, such as the unbiased two-stage method of moments approach using factor score approaches by Wall and Amemiya (2000, Wall & Amemiya, 2003), or further structure after measurement (SAM, Rosseel & Loh, 2022) methods such as the SAM-Croon (2002) approach by Cox & Kelcey (2021), or the the newly developed nonlinear local SEM (LSEM) approach by Rosseel et al. (2024) should be added if they are widely available.

Irmer et al. (2024) showed that the MSPE worked well even under distributional misspecification in QISEM using the UPI approach. Including features to model non-normal data would aid applied researchers to robustify their power analysis results. The results suggested that non-normal data resulted in higher required sample sizes. Hence, estimating power using the MSPE for QISEM under ideal circumstances will only estimate a lower bound for required sample sizes.

Also, methods specifically tailored to model non-normal data should be added such as the mixture modeling approach by Kelava et al. (2014), as they give applied researchers the opportunity to select the most adequate method for the distribution in the data.

Further, QISEM are more general models compared to linear SEM, as they include moderation, moderated mediation and quadratic effects. However, the class of nonlinear SEM is much larger and far more flexible. Hence, power estimation for more general nonlinear SEM procedures may also be possible using the MSPE approach and should be studied in future research.

As of now, we only considered cross sectional data. Hence, repeated measurements or clustered data have not been investigated. MSPE are possible in such scenarios, but the implementation in the powerNLSEM package does not include such features. The simulation of clustered or longitudinal data requires additional knowledge of even more parameters than are necessary for power analysis in QISEM, making it more challenging. Future research should consider power estimation for more complex estimation methods that also allow for nonlinear effects in data, such as the continuous time approaches or general hierarchical data methods that allow cross-level interaction effects. After further research on the applicability of the MSPE for these methods, extensions to multi-level data structures (Cox et al., 2023; Zyphur et al., 2019; McNeish & Hamaker, 2020), dynamical structural equation model (McNeish & Hamaker, 2020; Asparouhov et al., 2018) or to continuous time structural equation modeling (Voelkle et al., 2012) would make the MSPE and the powerNLSEM package widely usable.

Finally, all of the QISEM methods used in this paper were developed for continuous data. A large amount of data available in the social sciences consists of ordered-categorical data, e.g., by using Likert-type responses of questionnaires. However, research on linear SEM has shown that that ordered-categorical data cannot simply be treated as continuous (Foldnes & Grønneberg, 2022; Grønneberg & Foldnes, 2024; Rhemtulla et al., 2012), but strong assumptions need to be made (Foldnes & Grønneberg, 2022; Grønneberg & Foldnes, 2024). Simulation results on QISEM treating ordered-categorical data as continuous showed that asymmetry of thresholds results in large bias and inflated Type I error rates for LMS and UPI (Aytürk et al., 2020; Lodder et al., 2019). Hence, power estimation in such scenarios is affected by the inflated Type I error rates. Aytürk et al. (2021) examined an extension of LMS for ordered-categorical data which had an even longer runtime compared to LMS for continuous data. Future research is needed to examining the effects of ordered-categorical data in the context of QISEM on power estimation.

### Practical recommendations for power analysis using powerNLSEM

Power analysis for QISEM is complex as specific values for all parameters in the model need to be set. When theoretical or expected values are elusive, we suggest adopting the concept of Smallest Effect Sizes of Interest (SESOI, Anvari & Lakens, 2021). Moreover, our findings from Simulation Study 1 indicate that item reliability significantly impacts the required sample size for a method. When uncertainties arise regarding the selection of measurement parameters within the model, researchers should perform multiple power estimations using the MSPE approach across varying reliabilities. This strategy enables a wide search of how the required sample size varies with regard to reliability for the specific research scenario under consideration.

It is important to keep in mind that the MSPE procedure is simulation-based, introducing an element of sample variability. The risk of estimating a sample size smaller than the true size required for a desired power rate is dependent on the selected Type I error rate. Running the procedure several times will give slightly different results, with some sample sizes falling below the true requirement. If these deviations are substantial, we propose increasing the number of replications to enhance the stability of the probit regression estimation. Our simulations showed that $R=2000$ resulted in stable estimation of required sample sizes, however, in order to receive precise estimates of required sample sizes, much higher replication counts are necessary. We recommend to run at least $R=2000$ models and if time permits increase this number by a factor of 10-100. In Simulation Study 2 we used $R=10^5$ replications with good precision for the MSPE using the adaptive algorithm to select samples sizes. For the time-consuming method LMS, if the power analysis is not to be run for several, we recommend performing the MSPE with the adaptive algorithm several times with rather small replication numbers and using the largest estimated required sample size as this decreases the risk of using a sample size that is in fact too small with regard to the corresponding power rate. Further, performing power analysis using the UPI approach instead could be used with caution with high replication counts. The resulting required sample size could then be compared to the required sample sizes resulting from fewer replications for LMS.

Further, we suggest to inspect the solution of the powerNLSEM function. This can be achieved by employing the plot method on the resultant object, as exemplified in Fig. 2. To investigate the impact of the confidence interval width during the fitting step of the probit regression model within the reanalyse.powerNLSEM function, one can consider reducing the Type I error rate (argument alpha_power_modeling in the powerNLSEM or the reanalyse.powerNLSEM function).

## Conclusion

Conducting power analysis for nonlinear SEM poses considerable challenges. As we could demonstrate, the MSPE approach serves as a valuable instrument for modeling power in cases where analytical power solutions are absent, although sample sizes need to be selected in the fitting process. The newly proposed adaptive search algorithm dynamically selects sample sizes and therefore makes the MSPE method applicable to applied researchers. By being implemented in the powerNLSEM package, the MSPE method becomes accessible to applied researchers dealing with QISEM - a specialized form of nonlinear SEM. QISEM includes models that incorporate latent moderation, mediated moderation, and quadratic effects. Although the choice of parameters in complex models can be challenging, the potential to circumvent the wasting of resources by judiciously collecting data outweighs these challenges from an economic standpoint.

## Data Availability

Available at the OSF repository https://osf.io/k79hv.

## Appendix A Additional information on simulation studies

### A.1 Simulation conditions for the slimple latent moderation SEM

For the latent moderation model, we used two reliability conditions and used the following measurement models. Matrices are given in LISREL notation (Jöreskog, 1970).

#### A.1.1 High reliability

The following elements are rounded to 4 decimals.

$$\begin{aligned} \Lambda _x =\Lambda _y&= \begin{bmatrix} 1 & 0 \\ .8 & 0 \\ .7 & 0 \\ 0 & 1 \\ 0 & .8 \\ 0 & .7 \end{bmatrix}, \end{aligned}$$

$$\begin{aligned} \mathbb {C}ov \, \varvec{\xi } = \Phi = \begin{bmatrix} 1 & \\ \phi _{21} & 1 \end{bmatrix}&= \begin{bmatrix} 1 & \\ .5 & 1 \end{bmatrix}, \end{aligned}$$

for normally distributed $\varvec{\xi }=(\xi _1,\xi _2)'$ and $\varvec{\zeta }=(\zeta _1,\zeta _2)'$, where $\varvec{\zeta }$ and $\varvec{\xi }$ are independent.

$$\begin{aligned} \varvec{X}&= \Lambda _x \varvec{\xi } + \varvec{\delta },\\ \varvec{Y}&= \Lambda _y \varvec{\eta } + \varvec{\varepsilon }, \end{aligned}$$

with $\varvec{\xi }=(\xi _1,\xi _2)', \varvec{\eta }=(\eta _1,\eta _2)'$, and normally distributed $\varvec{\delta }$ and $\varvec{\varepsilon }$ with covariance matrices

$$\begin{aligned} \mathbb {C}ov \, \varvec{\delta } = \mathbb {C}ov \, \varvec{\varepsilon }&= \text {diag}(.2345, .36, .51,.2345, .36, .51), \end{aligned}$$

where $\text {diag}(v)$ creates a diagonal matrix with diagonal elements *v*. The measurement errors $\varvec{\delta }$ and $\varvec{\varepsilon }$ are independent to all other variables in the model. The corresponding item-level reliabilites were .81, .64, .49, .81, .64,  and .49 for the elements of $\varvec{X}$, respectively, and .81, .64, .49, .81, .64,  and .49 for $\varvec{Y}$, respectively.

#### A.1.2 Low reliability

The structural part was set as in the previous section. The following elements are rounded to 4 decimals.

$$\begin{aligned} \Lambda _x = \Lambda _y&= \begin{bmatrix} 1 & 0 \\ .6 & 0 \\ .5 & 0 \\ 0 & 1 \\ 0 & .6 \\ 0 & .5 \end{bmatrix},\\ \end{aligned}$$

for normally distributed $\varvec{\xi }=(\xi _1,\xi _2)'$ and $\varvec{\zeta }=(\zeta _1,\zeta _2)'$, where $\varvec{\zeta }$ and $\varvec{\xi }$ are independent.

$$\begin{aligned} \varvec{X}&= \Lambda _x \varvec{\xi } + \varvec{\delta },\\ \varvec{Y}&= \Lambda _y \varvec{\eta } + \varvec{\varepsilon }, \end{aligned}$$

with $\varvec{\xi }=(\xi _1,\xi _2)', \varvec{\eta }=(\eta _1,\eta _2)'$, and normally distributed $\varvec{\delta }$ and $\varvec{\varepsilon }$ with covariance matrices

$$\begin{aligned} \mathbb {C}ov \, \varvec{\delta } = \mathbb {C}ov \, \varvec{\varepsilon }&= \text {diag}(1.0408, .64, .75,1.0408, .64, .75). \end{aligned}$$

The measurement errors $\varvec{\delta }$ and $\varvec{\varepsilon }$ are independent to all other variables in the model. The corresponding item-level reliabilites were .49, .36, .25, .49, .36,  and .25 for the elements of $\varvec{X}$, respectively, and .49, .36, .25, .49, .36,  and .25 for $\varvec{Y}$, respectively.

#### A.1.3 Full model in lavaan notation

All coefficients are given in lavaan syntax in the following. The variances of the latent residuals were set in a way so that all latent variables have variances of 1 and that the exogeneous variables ($\xi _1$ and $\xi _2$) have zero mean. The model used in the high and low reliability condition are as follows.

### A.2 Conditions for the complex latent moderated mediation SEM

For the latent moderated mediation model, the measurement models correspond to the high reliability condition of the previous section. All coefficients are given in lavaan syntax in the following. The variances of the latent residuals were set in a way so that all latent variables have variances of 1 and that the exogeneous variables ($\xi _1$ and $\xi _2$) have zero mean.

The moderator effects were set to be .1, which translates to a variance increment of $1.25\%$ for the effect of $\xi _1\xi _2\rightarrow \eta _2$ and $2.85\%$ of $\xi _1\eta _1\rightarrow \eta _2$, if first the effect of $\xi _1\xi _2\rightarrow \eta _2$ is included into the model. If, on the other hand, the effect of $\xi _1\eta _1\rightarrow \eta _2$ is included first, this results in a variance increment of $1.2\%$ and the additional increment of $\xi _1\xi _2\rightarrow \eta _2$ then is $2.9\%$.

## Appendix C Additional Tables

**Table 3:** Descriptives on the Distribution of the Required Sample Sizes for the Low Reliability Condition for the Simple QISEM

Est	$\rho$	Search	Con	Ref	Mean	SD	Min	$q_{.05}$	Median	$q_{.95}$	Max	$\bar{N}_\text {lb}$	Type I error
LMS	0.50	Adaptive	200	207	206.5	19.2	145	174	207	233	253	240.0	0.030
LMS	0.50	Bruteforce	200	207	207.7	14.1	160	182	208	229	251	236.4	0.015
LMS	0.60	Adaptive	200	279	277.8	16.2	228	249	278	302	313	306.0	0.035
LMS	0.60	Bruteforce	200	279	278.8	13.3	235	256	279	298	320	306.2	0.015
LMS	0.70	Adaptive	200	367	366.0	12.6	331	345	366	386	396	389.2	0.030
LMS	0.70	Bruteforce	200	367	366.4	12.7	326	349	365	388	405	392.9	0.025
LMS	0.80	Adaptive	200	485	484.8	13.7	450	462	485	508	521	514.9	0.020
LMS	0.80	Bruteforce	200	485	484.3	13.9	439	463	485	508	531	513.6	0.020
LMS	0.90	Adaptive	200	676	677.5	30.0	619	634	677	731	784	750.6	0.010
LMS	0.90	Bruteforce	200	676	675.1	21.8	616	639	676	712	744	721.3	0.020
LMS	0.95	Adaptive	200	858	861.1	51.0	759	790	858	952	1,051	987.5	0.010
LMS	0.95	Bruteforce	200	858	856.5	32.9	771	805	856	911	951	927.6	0.020
UPI	0.50	Adaptive	200	221	221.0	20.7	152	189	222	253	273	256.8	0.020
UPI	0.50	Bruteforce	200	221	221.6	15.5	185	194	224	248	256	251.6	0.010
UPI	0.60	Adaptive	200	299	298.3	17.5	239	273	298	325	342	328.4	0.020
UPI	0.60	Bruteforce	200	299	298.3	14.8	262	273	299	322	330	326.9	0.005
UPI	0.70	Adaptive	200	394	393.9	13.6	353	372	394	418	425	419.1	0.020
UPI	0.70	Bruteforce	200	394	393.2	14.2	359	372	392	418	429	420.8	0.015
UPI	0.80	Adaptive	200	522	523.0	14.3	485	500	522	546	559	556.1	0.005
UPI	0.80	Bruteforce	200	522	520.9	15.7	483	497	519	548	568	552.2	0.025
UPI	0.90	Adaptive	200	730	732.4	31.3	667	681	730	784	827	811.8	0.015
UPI	0.90	Bruteforce	200	730	727.8	24.2	665	689	726	767	801	779.2	0.025
UPI	0.95	Adaptive	200	928	932.2	53.3	819	851	928	1,018	1,091	1,068.8	0.025
UPI	0.95	Bruteforce	200	928	924.9	36.3	827	866	925	981	1,029	1,004.6	0.025
FSR	0.50	Adaptive	200	207	205.8	19.2	145	175	207	235	246	240.9	0.035
FSR	0.50	Bruteforce	200	207	207.4	14.3	155	183	209	231	251	236.8	0.005
FSR	0.60	Adaptive	200	280	279.2	16.3	231	254	280	303	318	308.9	0.020
FSR	0.60	Bruteforce	200	280	280.5	13.6	232	258	280	304	318	308.5	0.010
FSR	0.70	Adaptive	200	371	370.5	12.7	340	351	370	391	406	394.8	0.005
FSR	0.70	Bruteforce	200	371	370.9	13.1	333	350	370	393	407	398.0	0.010
FSR	0.80	Adaptive	200	494	493.9	13.5	466	473	492	518	533	525.2	0.005
FSR	0.80	Bruteforce	200	494	492.9	14.7	453	471	493	514	545	522.9	0.020
FSR	0.90	Adaptive	200	692	694.5	29.5	627	651	693	744	800	771.8	0.015
FSR	0.90	Bruteforce	200	692	690.8	23.1	638	655	691	726	771	739.0	0.030
FSR	0.95	Adaptive	200	882	886.1	50.3	775	809	880	972	1,063	1,020.7	0.010
FSR	0.95	Bruteforce	200	882	879.5	34.8	793	825	880	943	988	954.3	0.020
SR	0.50	Adaptive	200	226	225.8	20.5	168	193	227	257	285	262.4	0.020
SR	0.50	Bruteforce	200	226	225.7	16.1	177	198	226	251	273	255.9	0.020
SR	0.60	Adaptive	200	307	306.5	17.3	254	278	306	332	355	337.7	0.015
SR	0.60	Bruteforce	200	307	306.2	15.3	264	282	306	330	356	334.9	0.025
SR	0.70	Adaptive	200	407	406.8	13.9	365	384	408	430	440	433.0	0.020
SR	0.70	Bruteforce	200	407	405.9	14.5	367	382	406	430	457	433.9	0.020
SR	0.80	Adaptive	200	542	542.3	15.7	502	515	542	565	588	576.7	0.030
SR	0.80	Bruteforce	200	542	540.6	15.5	502	514	540	567	591	573.3	0.030
SR	0.90	Adaptive	200	761	762.8	33.3	673	709	762	815	861	844.3	0.030
SR	0.90	Bruteforce	200	761	759.4	23.5	693	723	759	798	825	814.6	0.025
SR	0.95	Adaptive	200	970	973.3	55.5	833	885	967	1,065	1,133	1,113.3	0.035
SR	0.95	Bruteforce	200	970	968.2	35.6	860	912	970	1,027	1,057	1,054.1	0.025

*Note.* Est = estimation method, $\rho$ = power level, Search = search method, Con = number of replications the search method found a desired sample size, Ref = reference sample size based on $8\times 10^5$ replications, Mean, SD, Min, $q_{.05}$, Median, $q_{.95}$, and Max refer to $N_\alpha$, $q_x$ = x-quantile, $\bar{N}_\text {lb}$ = mean of required N for lower bound of power

**Table 4:** Descriptives on the Distribution of the Required Sample Sizes for the Low Reliability Condition for the Simple QISEM

Method	$\rho$	Search	Con	Ref	Mean	SD	Min	$q_{.05}$	Median	$q_{.95}$	Max	$\bar{N}_\text {lb}$	Type I error
LMS	0.50	Adaptive	200	516	514.4	30.5	440	465	514	564	588	568.9	0.020
LMS	0.50	Bruteforce	200	516	515.9	17.8	460	490	516	545	570	551.1	0.010
LMS	0.60	Adaptive	200	678	677.6	27.2	598	635	680	720	754	726.6	0.030
LMS	0.60	Bruteforce	200	678	677.6	20.0	623	648	676	712	748	720.6	0.005
LMS	0.70	Adaptive	200	877	877.3	26.5	793	834	878	918	956	926.2	0.025
LMS	0.70	Bruteforce	200	877	875.1	29.4	811	829	874	923	987	941.3	0.030
LMS	0.80	Adaptive	200	1142	1,143.8	35.0	1,050	1,088	1,144	1,202	1,245	1,212.8	0.030
LMS	0.80	Bruteforce	200	1142	1,138.4	47.7	1,024	1,069	1,138	1,215	1,309	1,247.9	0.040
LMS	0.90	Adaptive	200	1568	1,572.1	63.3	1,410	1,462	1,573	1,675	1,740	1,705.8	0.045
LMS	0.90	Bruteforce	200	1568	1,560.9	82.9	1,351	1,442	1,557	1,706	1,830	1,752.8	0.040
LMS	0.95	Adaptive	200	1970	1,977.3	96.7	1,731	1,812	1,976	2,140	2,236	2,187.3	0.040
LMS	0.95	Bruteforce	200	1970	1,960.1	119.3	1,656	1,779	1,954	2,169	2,326	2,237.3	0.045
UPI	0.50	Adaptive	200	977	978.6	39.9	886	910	977	1,052	1,085	1,053.8	0.005
UPI	0.50	Bruteforce	200	977	972.8	41.3	883	902	971	1,048	1,081	1,065.5	0.055
UPI	0.60	Adaptive	200	1230	1,231.7	35.8	1,152	1,175	1,229	1,299	1,330	1,302.5	0.015
UPI	0.60	Bruteforce	200	1230	1,223.4	64.8	1,085	1,120	1,216	1,339	1,429	1,373.0	0.060
UPI	0.70	Adaptive	200	1533	1,534.9	35.4	1,448	1,478	1,533	1,591	1,626	1,610.4	0.015
UPI	0.70	Bruteforce	200	1533	1,523.4	97.3	1,324	1,371	1,507	1,698	1,863	1,751.4	0.050
UPI	0.80	Adaptive	200	1931	1,932.4	45.6	1,802	1,860	1,932	2,004	2,036	2,034.9	0.020
UPI	0.80	Bruteforce	200	1931	1,916.4	144.0	1,624	1,701	1,892	2,163	2,445	2,256.8	0.050
UPI	0.90	Adaptive	200	2557	2,559.3	77.8	2,353	2,431	2,557	2,677	2,762	2,736.5	0.020
UPI	0.90	Bruteforce	200	2557	2,536.4	222.8	2,090	2,215	2,499	2,911	3,382	3,067.0	0.050
UPI	0.95	Adaptive	200	3141	3,143.4	115.5	2,834	2,960	3,136	3,328	3,445	3,406.7	0.020
UPI	0.95	Bruteforce	200	3141	3,113.9	300.1	2,518	2,688	3,063	3,641	4,271	3,831.4	0.050
FSR	0.50	Adaptive	200	574	579.6	33.5	488	522	580	629	653	643.7	0.015
FSR	0.50	Bruteforce	200	574	574.3	20.4	517	540	576	607	642	616.7	0.015
FSR	0.60	Adaptive	200	771	773.8	30.7	689	721	776	821	838	832.2	0.015
FSR	0.60	Bruteforce	200	771	775.5	26.8	714	730	775	819	856	836.6	0.010
FSR	0.70	Adaptive	200	1013	1,012.9	31.2	935	962	1,012	1,062	1,086	1,071.8	0.020
FSR	0.70	Bruteforce	200	1013	1,024.4	45.3	926	961	1,020	1,102	1,177	1,128.6	0.025
FSR	0.80	Adaptive	200	1339	1,333.8	42.1	1,213	1,265	1,336	1,391	1,441	1,416.5	0.055
FSR	0.80	Bruteforce	200	1339	1,359.8	77.3	1,186	1,235	1,355	1,495	1,629	1,537.2	0.020
FSR	0.90	Adaptive	200	1866	1,852.0	75.1	1,625	1,723	1,855	1,963	2,064	2,011.4	0.060
FSR	0.90	Bruteforce	200	1866	1,903.8	136.0	1,600	1,693	1,895	2,146	2,377	2,216.2	0.025
FSR	0.95	Adaptive	200	2367	2,344.1	113.5	1,996	2,148	2,343	2,521	2,674	2,594.4	0.060
FSR	0.95	Bruteforce	200	2367	2,422.1	196.2	1,989	2,126	2,408	2,770	3,101	2,873.4	0.020
SR	0.50	Adaptive	200	569	576.7	36.4	438	514	580	627	661	642.8	0.020
SR	0.50	Bruteforce	200	569	568.7	20.1	519	536	570	602	623	612.2	0.020
SR	0.60	Adaptive	200	771	775.9	32.5	658	721	777	823	858	836.1	0.025
SR	0.60	Bruteforce	200	771	775.9	26.4	702	734	776	821	841	839.2	0.010
SR	0.70	Adaptive	200	1022	1,021.9	30.3	939	976	1,021	1,076	1,103	1,082.5	0.010
SR	0.70	Bruteforce	200	1022	1,033.3	44.2	917	964	1,030	1,107	1,146	1,142.9	0.015
SR	0.80	Adaptive	200	1359	1,353.0	38.2	1,258	1,284	1,350	1,419	1,473	1,438.5	0.040
SR	0.80	Bruteforce	200	1359	1,381.5	75.3	1,199	1,269	1,378	1,510	1,583	1,569.7	0.020
SR	0.90	Adaptive	200	1906	1,889.3	70.5	1,685	1,768	1,892	1,995	2,137	2,055.1	0.040
SR	0.90	Bruteforce	200	1906	1,948.3	132.7	1,636	1,737	1,940	2,167	2,308	2,282.2	0.020
SR	0.95	Adaptive	200	2427	2,399.8	110.2	2,068	2,211	2,400	2,577	2,778	2,661.1	0.055
SR	0.95	Bruteforce	200	2427	2,489.8	191.6	2,048	2,187	2,480	2,791	3,008	2,973.9	0.015

*Note.* Est = estimation method, $\rho$ = power level, Search = search method, Con = number of replications the search method found a desired sample size, Ref = reference sample size based on $8\times 10^5$ replications, Mean, SD, Min, $q_{.05}$, Median, $q_{.95}$, and Max refer to $N_\alpha$, $q_x$ = x-quantile, $\bar{N}_\text {lb}$ = mean of required N for lower bound of power

**Table 5:** Descriptives on the Distribution of the Required Sample Sizes for the Complex QISEM for $R=2000$

Method	$\rho$	Search	Con	Ref	Mean	SD	Min	$q_{.05}$	Median	$q_{.95}$	Max	$\bar{N}_\text {lb}$	Type I error
LMS	0.50	Adaptive	100	489	495.1	37.1	403	427	504	546	567	561.5	0.020
LMS	0.60	Adaptive	100	654	659.3	31.0	574	605	664	697	726	716.2	0.020
LMS	0.70	Adaptive	100	858	861.2	25.4	789	824	861	898	918	911.8	0.020
LMS	0.80	Adaptive	100	1132	1,131.9	31.2	1,060	1,084	1,134	1,181	1,223	1,201.2	0.020
LMS	0.90	Adaptive	100	1574	1,569.1	65.3	1,418	1,472	1,570	1,671	1,741	1,720.1	0.040
LMS	0.95	Adaptive	100	1993	1,984.1	106.9	1,754	1,831	1,979	2,148	2,277	2,234.4	0.030
UPI	0.50	Adaptive	100	533	536.6	43.6	392	480	538	605	649	607.9	0.030
UPI	0.60	Adaptive	100	714	717.0	34.4	602	669	722	766	809	778.7	0.020
UPI	0.70	Adaptive	100	937	939.3	24.0	877	903	939	978	1,003	995.0	0.000
UPI	0.80	Adaptive	100	1237	1,238.0	29.8	1,156	1,201	1,234	1,286	1,338	1,314.0	0.010
UPI	0.90	Adaptive	100	1721	1,721.0	75.0	1,517	1,614	1,718	1,838	1,919	1,884.1	0.030
UPI	0.95	Adaptive	100	2180	2,180.1	127.8	1,853	2,002	2,178	2,374	2,561	2,449.3	0.050
FSR	0.50	Adaptive	100	313	315.8	26.7	252	277	311	357	388	368.1	0.000
FSR	0.60	Adaptive	100	422	423.6	22.4	371	391	421	460	479	467.5	0.000
FSR	0.70	Adaptive	100	557	556.8	18.1	518	526	555	591	604	592.5	0.000
FSR	0.80	Adaptive	100	739	736.1	21.4	679	707	734	773	811	781.0	0.020
FSR	0.90	Adaptive	100	1033	1,026.4	44.8	888	962	1,025	1,091	1,185	1,137.1	0.040
FSR	0.95	Adaptive	100	1313	1,302.8	73.7	1,082	1,201	1,303	1,418	1,546	1,495.4	0.040
SR	0.50	Adaptive	100	328	333.4	26.6	266	291	332	376	425	386.2	0.000
SR	0.60	Adaptive	100	442	446.1	22.6	390	411	447	481	531	490.6	0.000
SR	0.70	Adaptive	100	584	585.2	18.5	544	558	585	612	657	621.9	0.000
SR	0.80	Adaptive	100	776	772.1	21.4	722	736	774	803	842	818.9	0.040
SR	0.90	Adaptive	100	1085	1,074.4	43.6	973	1,001	1,078	1,138	1,243	1,186.4	0.040
SR	0.95	Adaptive	100	1380	1,361.8	71.9	1,208	1,250	1,365	1,465	1,634	1,554.9	0.050

*Note.* Est = estimation method, $\rho$ = power level, Search = search method, Con = number of replications the search method found a desired sample size, Ref = reference sample size based on $2\times 10^5$ replications for LMS and $102\times 10^5$ replications for UPI, FSR, and SR; Mean, SD, Min, $q_{.05}$, Median, $q_{.95}$, and Max refer to $N_\alpha$ $q_x$ = x-quantile, $\bar{N}_\text {lb}$ = mean of required N for lower bound of power

**Table 6:** Descriptives on the Distribution of the Required Sample Sizes for the Complex QISEM for $R=10^5$

Method	$\rho$	Search	Con	Ref	Mean	SD	Min	$q_{.05}$	Median	$q_{.95}$	Max	$\bar{N}_\text {lb}$	Type I error
UPI	0.50	Adaptive	100	533	533.0	6.2	522	524	533	542	551	543.9	0.020
UPI	0.60	Adaptive	100	714	714.1	5.1	705	706	714	722	728	723.3	0.020
UPI	0.70	Adaptive	100	937	937.2	3.9	930	931	937	943	948	944.9	0.020
UPI	0.80	Adaptive	100	1237	1,236.6	4.5	1,226	1,230	1,236	1,244	1,247	1,246.7	0.005
UPI	0.90	Adaptive	100	1721	1,720.5	10.1	1,697	1,706	1,722	1,735	1,745	1,741.6	0.015
UPI	0.95	Adaptive	100	2180	2,180.4	17.0	2,141	2,155	2,182	2,205	2,219	2,214.4	0.025
FSR	0.50	Adaptive	100	313	313.0	5.0	297	304	313	320	327	321.6	0.035
FSR	0.60	Adaptive	100	422	422.2	4.1	410	414	423	428	434	429.3	0.020
FSR	0.70	Adaptive	100	557	557.1	3.0	551	552	557	562	565	562.4	0.005
FSR	0.80	Adaptive	100	739	738.7	3.1	732	734	739	743	749	744.7	0.005
FSR	0.90	Adaptive	100	1033	1,033.0	7.5	1,017	1,021	1,032	1,044	1,056	1,047.4	0.015
FSR	0.95	Adaptive	100	1313	1,313.2	13.0	1,287	1,291	1,312	1,333	1,356	1,337.8	0.010
SR	0.50	Adaptive	100	328	327.6	4.6	317	320	328	335	340	336.5	0.020
SR	0.60	Adaptive	100	442	442.4	3.8	434	436	442	449	452	449.6	0.015
SR	0.70	Adaptive	100	584	584.4	2.9	578	579	584	589	591	589.8	0.020
SR	0.80	Adaptive	100	776	775.5	3.1	768	771	776	780	783	781.9	0.030
SR	0.90	Adaptive	100	1085	1,085.4	6.9	1,071	1,074	1,086	1,095	1,102	1,100.5	0.030
SR	0.95	Adaptive	100	1380	1,380.6	11.8	1,352	1,361	1,381	1,397	1,408	1,406.2	0.035

*Note.* Est = estimation method, $\rho$ = power level, Search = search method, Con = number of replications the search method found a desired sample size, Ref = reference sample size based on $102\times 10^5$ replications, Mean, SD, Min, $q_{.05}$, Median, $q_{.95}$, and Max refer to $N_\alpha$, $q_x$ = x-quantile, $\bar{N}_\text {lb}$ = mean of required N for lower bound of power

## Appendix D Power Estimation using powerNLSEM for LMS, UPI, FSR, and SR

In this section we display the code to conduct the analysis of the main article using LMS, UPI, FSR, and SR.

The ggarrange function of the ggpubr package (Kassambara, 2023) stacks the ggplot (Wickham, 2016) plots produced by plot(LMS_adaptive, se = TRUE), plot(UPI_adaptive, se = TRUE), plot(FSR_a daptive, se = TRUE), and plot(SR_adaptive, se = TRUE) into a single plot and uses a common legend (common.legend = TRUE).

## Appendix E Information on R-packages used in the simulations

All empirical analyses were done in R (R Core Team, 2023). The main package of the article was the powerNLSEM package (Irmer, 2024), which uses the lavaan (Rosseel, 2012) for data handling and the fitting of UPI, FSR, and SR. LMS was called via MplusAutomation (Hallquist & Wiley, 2018) within the powerNLSEM package. ggplot (Wickham, 2016) is called to plot results.

We further utilized the papaja package for easy table handling (Aust & Barth, 2022).

Table 7 shows the corresponding package versions. The version number corresponding to the base package corresponds to the R version used.

**Table 7:** R Packages

Package	Version
base	4.3.1
datasets	4.3.1
ggplot2	3.5.0
graphics	4.3.1
grDevices	4.3.1
lavaan	0.6.17
methods	4.3.1
MplusAutomation	1.1.1
papaja	0.1.1
parallel	4.3.1
pbapply	1.7.2
powerNLSEM	0.1.0
stats	4.3.1
tinylabels	0.2.3
utils	4.3.1

*Note.* Implicitly loaded packages are also displayed

## Footnotes

1 2019 16-inch MacBook Pro with an 6-Core Intel Core i7 processor @2.6 GHz with 12 logical cores and 16 GB RAM

2 Shared computer cluster with 2 sockets of AMD EPYC 7452 32-core Processors @2.35 GHz (64 phyiscal cores and 128 logical cores) and 128GB RAM

3 Shared computer cluster with 2 sockets of AMD EPYC 7452 32-core Processors @2.35 GHz (64 phyiscal cores and 128 logical cores) and 128GB RAM

## Code Availability

Available at the OSF repository https://osf.io/k79hv. Description of the powerNLSEM package can be found at https://github.com/jpirmer/powerNLSEM.
